# CUL4B Promotes Breast Carcinogenesis by Coordinating with Transcriptional Repressor Complexes in Response to Hypoxia Signaling Pathway

**DOI:** 10.1002/advs.202001515

**Published:** 2021-03-16

**Authors:** Wei Huang, Jingyao Zhang, Miaomiao Huo, Jie Gao, Tianshu Yang, Xin Yin, Pei Wang, Shuai Leng, Dandan Feng, Yang Chen, Yang Yang, Yan Wang

**Affiliations:** ^1^ Beijing Key Laboratory of Cancer Invasion and Metastasis Research Advanced Innovation Center for Human Brain Protection Department of Biochemistry and Molecular Biology School of Basic Medical Sciences Capital Medical University Beijing 100069 China; ^2^ State Key Laboratory of Molecular Oncology National Cancer Center National Clinical Research Center for Cancer Cancer Hospital Chinese Academy of Medical Sciences and Peking Union Medical College Beijing 100021 China; ^3^ Collaborative Innovation Center of Tianjin for Medical Epigenetics Tianjin Key Laboratory of Medical Epigenetics Key Laboratory of Immune Microenvironment and Disease (Ministry of Education) Department of Biochemistry and Molecular Biology School of Basic Medical Sciences Tianjin Medical University Tianjin 300070 China

**Keywords:** breast cancer, cancer stem cell, CUL4B, epithelial‐mesenchymal transition, HIF1A

## Abstract

Cullin4B (CUL4B) is a scaffold protein of the CUL4B‐Ring E3 ligase (CRL4B) complex. However, the role of CUL4B in the development of breast cancer remains poorly understood. Here it is shown that CRL4B interacts with multiple histone deacetylase (HDAC)‐containing corepressor complexes, including MTA1/NuRD, SIN3A, CoREST, and NcoR/SMRT complexes. It is demonstrated that CRL4B/NuRD(MTA1) complexes cooccupy the E‐cadherin and AXIN2 promoters, and could be recruited by transcription factors including Snail and ZEB2 to promote cell invasion and tumorigenesis both in vitro and in vivo. Remarkably, CUL4B responded to transformation and migration/invasion stimuli and is essential for multiple epithelial‐mesenchymal transition (EMT) signaling pathways such as hypoxia. Furthermore, the transcription of CUL4B is directedly activated by hypoxia‐inducible factor 1*α* (HIF1*α*) and repressed by the ER*α*‐GATA3 axis. Overexpressing of CUL4B successfully induced CSC‐like properties. Strikingly, CUL4B expression is markedly upregulated during breast cancer progression and correlated with poor prognosis. The results suggest that CUL4B lies at a critical crossroads between EMT and stem cell properties, supporting CUL4B as a potential novel target for the development of anti‐breast cancer therapy.

## Graphical abstract

Cullin4B (CUL4B) is identified as a master regulator in promoting breast cancer carcinogenesis, metastasis, and stemness. The CUL4B‐Ring E3 ligase/NuRD (MTA1) complexes repress ER*α*, E‐cadherin, and AXIN2 through epigenetic regulatory mechanisms. The transcription of CUL4B is stimulated by HIF‐1*α* and suppressed by the ER*α*‐GATA3 axis. These findings support the pursuit of CUL4B as a promising therapeutic target for breast cancer.

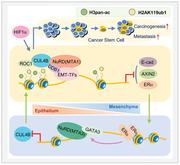

## 1 Introduction

Cullin‐Ring E3 ligase (CRL) complexes represent the largest family of ubiquitin E3 ligases and participate in a wide array of physiologically and developmentally controlled processes including cell cycle progression, replication, and the DNA damage response.^[^ ^1^, ^2^ ^]^ CRL4, a member of the CRL family, binds to a small RING finger protein, either ROC1 or ROC2, to recruit an E2 ubiquitin‐conjugating enzyme through its C‐terminal domain, and utilizes damaged DNA binding protein 1 (DDB1) as a linker protein to recruit various substrate‐recognition proteins.^[^ ^3^, ^4^ ^]^ In mammals, CUL4 consists of two cullin members, CUL4A and CUL4B, which share up to 82% protein sequence identity. Among them, CUL4B‐Ring E3 ligase (CRL4B) complex is composed of CUL4B, DDB1 and ROC1 as the core components. We have previously shown that the CRL4B complex regulates transcription by monoubiquitinating histone H2AK119 and by coordinating with polycomb repressive complex 2 (PRC2) or with the SUV39H1/HP1/DNMT3A complex to catalyze DNA methylation.^[^ ^5^, ^6^ ^]^

The histone acetylation state is balanced by two enzyme families: histone acetyltransferases (HATs) and histone deacetylases (HDACs).^[^ ^7^, ^8^ ^]^ Histone deacetylation is thought to facilitate chromosomal condensation, resulting in gene transcription repression.^[^ ^9^ ^]^ The zinc‐dependent HDACs traditionally comprise eleven members in vertebrates, referred to as HDAC1–11, which are further divided into classes I, II, and IV.^[^ ^10^, ^11^ ^]^ HDAC1, HDAC2, HDAC3, and HDAC8 are all class I HDACs.^[^ ^12^ ^]^ In mammals, class I HDACs are usually found in four multiprotein complexes: the metastasis‐associated 1/nucleosome remodeling and deacetylation (MTA1/NuRD) complex, the SIN3A complex, the CoREST complex, and the nuclear receptor corepressor (NcoR)/SMRT complex.^[^ ^13^ ^]^ The NuRD complex is composed of six core subunits and activates two known enzymatic pathways: ATP‐dependent chromatin remodeling activity by Mi‐2*α*/*β* and histone deacetylation; the latter is performed by HDAC1 and HDAC2.^[^ ^14^, ^15^ ^]^ The other subunits are methyl‐CpG‐binding domain 2 (MBD2) and MBD3, retinoblastoma‐binding protein 4 (RBBP4) and RBBP7, and metastasis‐associated gene 1/2/3 (MTA1/2/3).^[^ ^16^ ^]^ Interestingly, it has been confirmed that the vertebrate MTA family members MTA1, MTA2, and MTA3 are all affiliated with the NuRD complex, even though they have functionally unique features.^[^ ^17^ ^]^ MTA3 is an estrogen‐dependent component of the Mi‐2/NuRD transcriptional corepressor in breast epithelial cells.^[^ ^18^ ^]^ MTA1 is a potent corepressor of estrogen responsive element (ERE) transcription, as it blocks the ability of estradiol to stimulate estrogen receptor (ER)‐mediated transcription.^[^ ^19^ ^]^ Previously, we investigated the molecular mechanism of the opposing actions between MTA1 and MTA3 in breast cancer.^[^ ^20^ ^]^

The epithelial‐mesenchymal transition (EMT) is a multistep process of dynamic changes that is critical during morphogenesis and plays an indispensable role in tumor progression.^[^ ^21^, ^22^ ^]^ A series of transcription factors including SNAI1 (Snail), SLUG (Slug), TWIST1 (Twist), E47, and ZEB1/2 repress the expression of E‐cadherin to trigger EMT progression by recruiting diverse histone‐modification complexes.^[^ ^23^ ^]^ EMT can be triggered by multiple cellular signaling pathways including transforming growth factor‐*β* (TGF‐*β*), Notch, and Wnt.^[^ ^24^, ^25^ ^]^ Cancer stem cells (CSCs) have been identified in several solid tumors^[^ ^26^, ^27^, ^28^ ^]^ and are considered directly associated with treatment resistance and relapse following therapy.^[^ ^29^, ^30^ ^]^ CD44^+^/CD24^–/low^ and aldehyde dehydrogenase (ALDH) have been reported as markers for breast CSCs.^[^ ^26^, ^31^, ^32^ ^]^ Increasing evidence indicates that EMT progression plays an essential role in the CSC phenotype,^[^ ^33^, ^34^, ^35^, ^36^, ^37^ ^]^ suggesting that the EMT may be a therapeutic target for inhibiting CSCs to treat breast cancer. The downstream transcription factors of several cellular signaling pathways, including TGF*β*, Wnt, Hedgehog, and Notch, also act as cofactors to induce and maintain the CSC state.^[^ ^38^ ^]^

In this study, we investigated the role of CRL4B in the development of breast cancer. We explored CRL4B‐interacting proteins, genomic targets, and potential signaling pathways involved in breast cancer progression. We found that CRL4B interacts with massive transcriptional repressor complexes and is essential for the induction of EMT in breast carcinogenesis and thereby the generation of the CSCs.

## 2 Results

### 2.1 Cullin 4B‐RING E3 Ligase is Physically Associated with HDAC‐Containing Complexes

**Figure 1:**
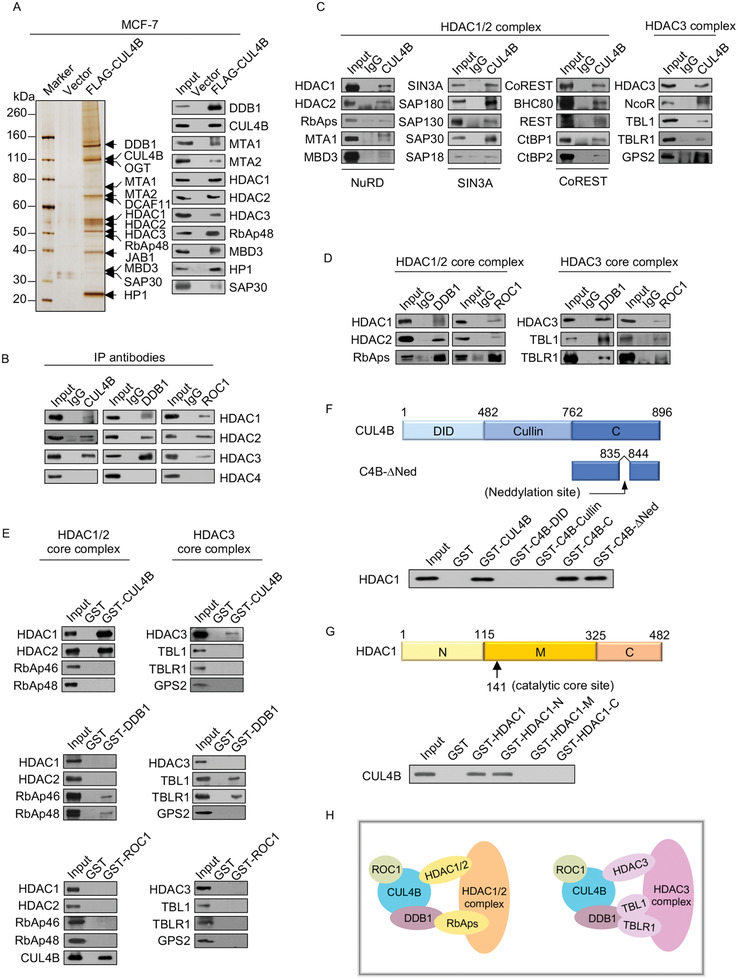
Cullin 4B‐RING E3 ligase is physically associated with HDAC‐containing complexes. A) Immunoaffinity purification and mass spectrometry analysis of CUL4B‐associated proteins. Whole‐cell extracts were prepared from FLAG‐CUL4B‐expressing MCF‐7 cells, subjected to affinity purification with anti‐FLAG affinity columns, and eluted with FLAG peptide. The eluates were resolved by SDS‐PAGE and silver‐stained (left). Purified fractions were analyzed by western blotting using the indicated antibodies (right). B) Immunoprecipitation assays in MCF‐7 cells with anti‐CRL4B followed by immunoblotting with antibodies against the HDAC1–4 proteins. C,D) Association of CRL4B with four multiprotein complexes in MCF‐7 cells. Whole‐cell lysates from MCF‐7 cells were prepared and immunoprecipitation was performed with anti‐CUL4B, anti‐DDB1, or anti‐ROC1 followed by immunoblotting with antibodies against the indicated proteins. E) Molecular interactions between CRL4B and core subunits of HDAC1/2/3 complexes. GST pull‐down assays were performed with bacterially expressed GST‐fused proteins and the indicated in vitro transcribed/translated proteins. F,G) Identification of the essential domains required for the interaction between CUL4B and HDAC1. C4B, CUL4B; Ned, Neddylation. H) Schematic representation of the interaction between CRL4B and corepressor complexes.

To determine the regulatory mechanisms of the CRL4B complex in breast cancer carcinogenesis, we analyzed proteins associated with CUL4B in human breast cancer adenocarcinoma MCF‐7 cells. Mass spectrometric analysis indicated that CUL4B was copurified with DDB1, MTA1, MTA2, HDAC1, HDAC2, HDAC3, RbAp48, MBD3, SAP30, and heterochromatin protein 1 (**Figure** **1**A, left). Some of these results were reported previously, such as DDB1,^[^ ^39^ ^]^ JAB1,^[^ ^40^ ^]^ and heterochromatin protein 1.^[^ ^6^ ^]^ The presence of these proteins in the CUL4B‐associated complex was confirmed by western blotting analysis of the column eluates with antibodies against the corresponding proteins (Figure 1A, right). To confirm the association between CUL4B and HDACs, coimmunoprecipitation experiments were performed with specific antibodies to detect endogenous proteins using total proteins derived from MCF‐7 cells. Immunoprecipitation with antibodies against CUL4B, DDB1, and ROC1, followed by immunoblotting with antibodies against HDAC1‐4, showed that CUL4B/DDB1/ROC1 more readily coimmunoprecipitated with HDAC1/2/3 (Figure 1B). The detailed results of mass spectrometric analysis are shown in Table S1 (Supporting Information 1).

To verify these results, we transfected MCF‐7 cells with GAL4‐CUL4B using three different luciferase reporter systems and treated the cells with or without trichostatin A, an inhibitor of class I and II HDACs.^[^ ^41^ ^]^ The results indicated that CUL4B markedly repressed the activities of all three reporter gene systems in a dose‐dependent manner (Figure S1A, Supporting Information 1). Overexpression of FLAG‐CUL4B had little effect on the activities of reporter genes, indicating that the repression of luciferase activities caused by GAL4‐CUL4B occurred because of specific recruitment of CUL4B in the promoter region of reporter genes. Additionally, trichostatin A treatment strongly counteracted the repression of reporter gene activity by activating the expression of GAL4‐CUL4B (Figure S1B, Supporting Information 1). Together, these results confirm that CUL4B‐mediated transcription repression is linked to HDAC activity.

The NuRD, SIN3A, CoREST, and NcoR/SMRT complexes are the four main HDAC‐containing complexes.^[^ ^13^ ^]^ We next validated the interactions between CRL4B and the four multiprotein complexes mentioned above using total proteins derived from MCF‐7 cells. Immunoprecipitation with antibodies against CUL4B was followed by immunoblotting with antibodies against the HDAC1, HDAC2, RbAp46/48, MTA1, and MBD3 subunits of the NuRD complex; SIN3A, SAP180, SAP130, SAP30, and SAP18 subunits of the SIN3A complex; CoREST, BHC80, REST, CTBP1, and CTBP2 subunits of the CoREST complex; and HDAC3, NCoR, TBL1, TBLR1, and GPS2 subunits of the NcoR/SMRT complex. All tested proteins were efficiently coimmunoprecipitated with CUL4B (Figure 1C). Furthermore, immunoprecipitation with antibodies against DDB1 or ROC1 and immunoblotting with antibodies against HDAC1, HDAC2, RbAp46/48, HDAC3, TBL1, or TBLR1 indicated a strong association of the HDAC complexes with DDB1 and ROC1 (Figure 1D). Moreover, fast protein liquid chromatography experiments showed that the native CUL4B from MCF‐7 cells was eluted at an apparent molecular mass that is much greater than that of the monomeric protein; CUL4B immunoreactivity was detected in chromatographic fractions from the Superose 6 column with a peak range of 669‐2000 kDa. Significantly, overlapping elution patterns were detected between CUL4B and components of the corepressor complexes in corresponding fractions (Figure S1C, Supporting Information 1), supporting that CRL4B is associated with multiple corepressor complexes.

To further explore the molecular basis of the interaction between CRL4B and these HDAC complexes, we performed glutathione *S*‐transferase (GST) pull‐down assays using GST‐fused CUL4B and in vitro transcribed/translated components of the corepressor complexes. The results showed that CUL4B interacted with HDAC1, HDAC2, and HDAC3 (Figure 1E, upper panels), whereas DDB1 interacted with RbAp46, RbAp48, TBL1, and TBLR1, four WD40‐containing molecules (Figure 1E, middle panels). In contrast, ROC1 did not interact with these molecules (Figure 1E, lower panels). These results suggest that DDB1 and its DWD motif act as a bridge to support the interactions between the two core enzyme complexes, CUL4B and HDACs. Moreover, to further mapping which domains mediated the interactions of CUL4B between HDAC1, GST pull‐down assays were performed with a series of truncation vectors (Figure 1F,G, upper panels). The results showed that the C terminal of CUL4B and the N terminal of HDAC1 were essential for the direct interaction (Figure 1F,G, lower panels). Together, these experiments revealed the molecular mechanisms involved in the interaction of the CRL4B complex with multiple corepressor complexes and provide additional insight into the mechanisms underlying the physical association between CUL4B/DDB1/ROC1 and these corepressor complexes (Figure 1H).

### 2.2 Identification of Genome‐Wide Transcription Targets for CRL4B and Its Associated Corepressor Complexes

**Figure 2:**
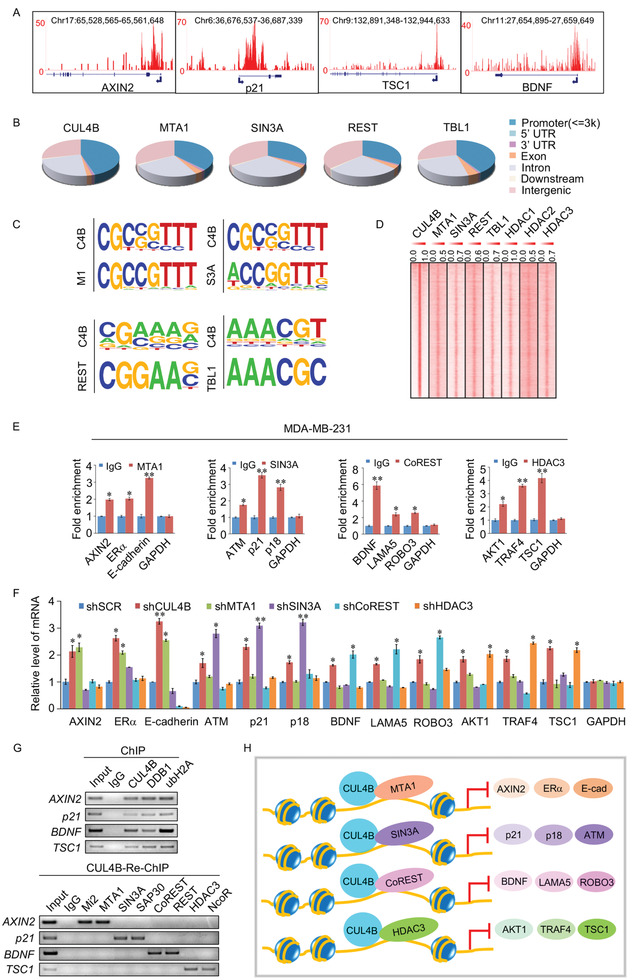
Identification of genome‐wide transcription targets of the CRL4B complex. A) Binding profiles of CUL4B on the representative target genes AXIN2, p21, TSC1, and BDNF. The chromosome number and position of the peak bound by each protein are shown. B) Genomic distribution of CUL4B, MTA1, SIN3A, REST, and TBL1 determined by ChIP‐seq analysis. C) MEME analysis of the DNA‐binding motifs of CUL4B, MTA1, SIN3A, REST, and TBL1. C4B, CUL4B; M1, MTA1; S3A, SIN3A. D) ChIP‐seq density heatmaps of CUL4B, MTA1, SIN3A, REST, TBL1, HDAC1, HDAC2, and HDAC3. E) Verification of the ChIP‐seq results through qChIP analysis of the indicated genes in MDA‐MB‐231 cells. Results are represented as fold change relative to control with GAPDH as a negative control. F) qRT‐PCR measurement of the expression of the indicated genes selected from ChIP‐seq results in MDA‐MB‐231 cells under depletion of CUL4B, MTA1, SIN3A, CoREST, or HDAC3 using lentivirus‐delivered small hairpin RNA (shRNA). G) ChIP and ChIP/Re‐ChIP experiments on the promoter of the indicated genes with antibodies against the indicated proteins in MDA‐MB‐231 cells. ubH2A, H2AK119ub1. H) Schematic representation of the interactions between CUL4B and the NuRD, SIN3A, REST/CoREST, and NCoR/SMRT corepressor complexes that repress the expression of different sets of genes. E,F) Error bar represents mean ± SD of three independent experiments. *p < 0.05, **p < 0.01. Student's t‐test.

To better investigate the biological significance of the CRL4B/HDAC complex, we analyzed the occupancy of genome‐wide transcriptional targets of CUL4B. Chromatin immunoprecipitation^[^ ^42^ ^]^ was conducted first in MDA‐MB‐231 cells with antibodies against CUL4B, and then CUL4B‐associated DNA sequences were amplified for chromatin immunoprecipitation‐based deep sequencing (ChIP‐seq), library construction, cluster generation, and sequencing using an Illumina HiSeq2000. Using model‐based analysis of ChIP‐Seq software, we detected 36000 CUL4B‐specific binding peaks representing the ChIP‐seq peak (**Figure** **2**A) (GEO accession number: GSE124611). We next performed cross‐analysis of DNA promoter sequences that overlapped with publicly available ChIP‐seq datasets for MTA1 (GSE91687), SIN3A (GSM1010862), REST (GSM1010891), and TBL1 (GSM865743); these sequences represented co‐targets of the CRL4B/NuRD, CRL4B/SIN3A, CRL4B/CoREST, and CRL4B/NCoR/SMRT complexes, respectively (Figure 2B).

Notably, analysis of the genomic signatures showed similar DNA binding motifs between CUL4B and MTA1, CUL4B and SIN3A, CUL4B and REST, and CUL4B and TBL1 (Figure 2C). The distribution of binding loci relative to the transcription start sites of CUL4B, MTA1, SIN3A, REST, and TBL1 is shown in Figure S2A, Supporting Information 1. Heatmap visualization of the promoter peaks was performed using ChIPseeker (Figure S2B, Supporting Information 1).^[^ ^43^ ^]^ Additionally, comparisons of the characteristic enrichment of CUL4B, MTA1, SIN3A, REST, and TBL1, as well as HDAC1 (GSM2828500), HDAC2 (GSM2423339), and HDAC3 (GSM2302871), indicated that these proteins were significantly enriched in regions surrounding the CUL4B‐binding sites (Figure 2D).

The promoters targeted by CUL4B, MTA1, SIN3A, REST, and TBL1 included 7043 promoters targeted by CUL4B and MTA1, 6901 promoters targeted by CUL4B and SIN3A, 5324 promoters regulated by CUL4B and REST, and 7575 promoters targeted by CUL4B and TBL1 (Figure S2C, upper panels, Supporting Information 1). These promoters were analyzed using the Database for Annotation, Visualization and Integrated Discovery bioinformatics resource 6.8 and classified into various cellular signaling pathways, including cell cycle, MAPK, focal adhesion, p53, hypoxia, Wnt, TGF‐*β*, Notch, signaling pathways regulating pluripotency of stem cells, and estrogen pathways (Figure S2C, lower panel, Supporting Information 1). To verify the ChIP‐seq results, quantitative ChIP (qChIP) analysis was performed on MDA‐MB‐231 cells using specific antibodies against MTA1, SIN3A, CoREST, and HDAC3 targeting their indicated distinct target genes (Figure 2E). The results showed that CUL4B, MTA1, SIN3A, CoREST, and TBL1 bound to the promoters of their representative genes, validating the ChIP‐seq results. Moreover, quantitative real‐time PCR (qRT‐PCR) assays showed the mRNA expression levels of the common target genes in each group were significantly increased in response to knockdown treatments targeting the corresponding subunits without affecting those in other groups (Figure 2F). To substantiate the hypothesis that CRL4B nucleates these four corepressor complexes to regulate unique target genes, we chose four representative target genes, *AXIN2*, *p21*, *BDNF*, and *TSC1* corresponding to each of the four complexes, for further verification by sequential ChIP or ChIP/Re‐ChIP experiments. Soluble chromatins were immunoprecipitated with antibodies against CUL4B, DDB1, ROC1, and H2AK119ub1 (Figure 2G, upper panels), indicating that these four target genes were repressed by CRL4B through promoter H2AK119ub1 modification, a major histone modification marker for transcriptional repression.^[^ ^5^ ^]^ The first‐round ChIP elutes were then reimmunoprecipitated with the appropriate antibodies and data showed that each representative promoter was only reimmunoprecipitated with the corresponding antibodies of each complex, respectively (Figure 2G, lower panels). These results indicate that CUL4B regulates specific target genes through functional coordination with specific HDAC‐containing complexes (Figure 2H). Detailed results of the ChIP‐seq experiments were provided in Supporting Information 2 and deposited in GEO data sets.

### 2.3 CRL4B is Physically Associated with the NuRD(MTA1) Complex

**Figure 3:**
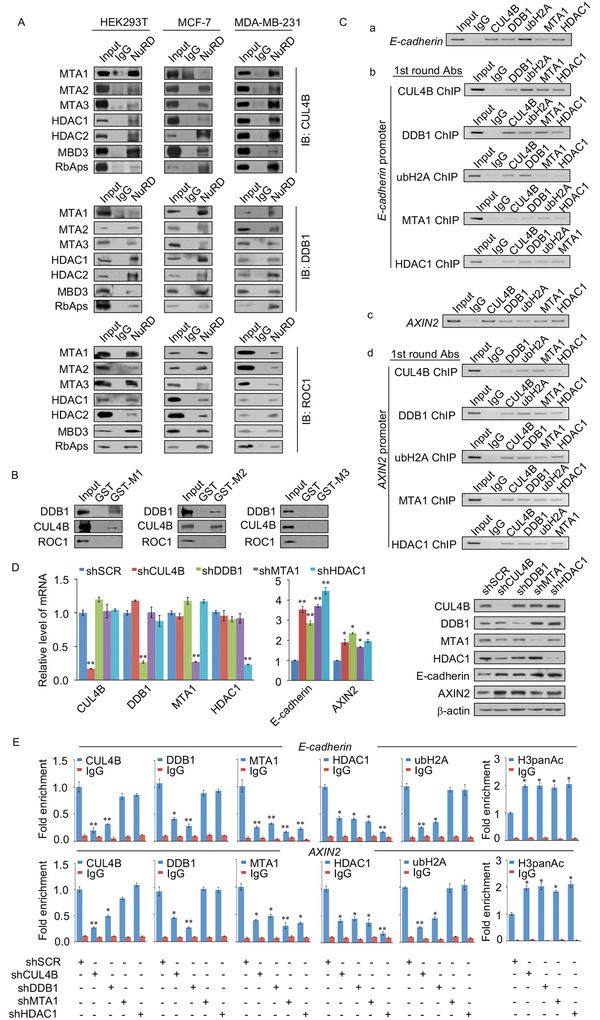
CRL4B is physically associated with the NuRD(MTA1) complex. A) Association of CRL4B with the NuRD(MTA1) complex. Whole‐cell lysates from HEK293T cells (left), MCF‐7 cells (middle), or MDA‐MB‐231 cells (right) were prepared and immunoprecipitated with antibodies against the indicated proteins. Immunocomplexes were then immunoblotted using antibodies against CUL4B, DDB1, and ROC1. B) GST pull‐down experiments were performed with GST‐fused MTA1/2/3 and in vitro transcribed/translated components of the CRL4B complex. M1, MTA1; M2, MTA2; M3, MTA3. C) The CRL4B/NuRD(MTA1) complex was found in the same protein complex on the *E‐cadherin* (a,b) and *AXIN2* (c,d) promoters. ChIP and Re‐ChIP experiments were performed in MDA‐MB‐231 cells with the indicated antibodies. ubH2A, H2AK119ub1. D) MDA‐MB‐231 cells were infected with lentivirus carrying control shRNA (shSCR) or shRNAs targeting CUL4B, DDB1, MTA1, or HDAC1. The knockdown efficiencies of CUL4B, DDB1, MTA1, and HDAC1 were confirmed by RT‐PCR (left panels) and western blotting (right panels), expression levels of the target genes, E‐cadherin and AXIN2, were also be analyzed. E) MDA‐MB‐231 cells were infected with lentiviruses carrying the indicated shRNAs. qChIP analysis of the recruitment of the *E‐cadherin* and *AXIN2* promoters was performed using the indicated antibodies. Purified rabbit IgG was used as a negative control. ubH2A, H2AK119ub1. D,E) Error bar represents mean ± SD of three independent experiments. *p < 0.05, **p < 0.01. Student's t‐test.

As described above, CUL4B was copurified with MTA1, MTA2, HDAC1, HDAC2, RbAp48, and MBD3; we then found preliminary evidence that CUL4B is physically associated with the NuRD complex. To further validate these interactions, total proteins from HEK293T, MCF‐7, and MDA‐MB‐231 cells were extracted, and coimmunoprecipitation experiments were performed (**Figure** **3**A). The reciprocal coimmunoprecipitation results illustrate that CRL4B is physically associated with the NuRD complex. We next examined the molecular basis of the interaction between CUL4B and the NuRD complex. GST‐fused MTA1, MTA2, or MTA3 proteins were purified and incubated with in vitro transcribed/translated components of the CRL4B complex containing CUL4B, DDB1, and ROC1. Unexpectedly, CUL4B and DDB1 only directly interacted with MTA1 and MTA2, whereas neither interacted with MTA3 (Figure 3B). In addition, further GST pull‐down assays demonstrated that CUL4B interacted with HDAC1 and HDAC2, with DDB1 showing additional direct interactions with RbAp46 or RbAp48 (Figure 1F). These results support the specific interaction mechanisms between the CRL4B and NuRD complexes (Figure S3A, Supporting Information 1).

Of the three members of the MTA family, MTA1 is the most strongly correlated with tumor malignancy.^[^ ^44^ ^]^ To further investigate whether CRL4B and NuRD(MTA1) form a complex to bind target gene promoters, sequential ChIP or ChIP/Re‐ChIP experiments were performed in MDA‐MB‐231 cells. Soluble chromatins were immunoprecipitated with antibodies against CUL4B, DDB1, H2AK119ub1, MTA1, and HDAC1 (Figure 3C‐a,c). The immunoprecipitates were then reimmunoprecipitated with appropriate antibodies. The results showed that *E‐cadherin* and *AXIN2* promoters immunoprecipitated with antibodies against CUL4B were reimmunoprecipitated with antibodies against DDB1, MTA1, HDAC1, and H2AK119ub1 (Figure 3C‐b,d). Similar results were obtained when initial ChIP was performed with antibodies against DDB1, MTA1, HDAC1, and H2AK119ub1 (Figure 3C‐b,d). These results support that CRL4B and the NuRD(MTA1) complex occupy the *E‐cadherin* and *AXIN2* promoters as a coordinated complex.

To investigate the functional relationship between CRL4B and the MTA1/NuRD complex on *E‐cadherin* and *AXIN2* promoters, MDA‐MB‐231 cells were treated with specific short hairpin RNAs (shRNAs) to stably deplete CUL4B, DDB1, MTA1, or HDAC1. As expected, depletion of each factor in MDA‐MB‐231 cells resulted in increased expression of E‐cadherin and AXIN2 at both the mRNA and protein levels (Figure 3D). qChIP assays showed that suppression of CUL4B or DDB1 expression resulted in a significant reduction in the recruitment of MTA1 and HDAC1 to the *E‐cadherin* and *AXIN2* promoters, whereas MTA1 or HDAC1 depletion had little effect on the recruitment of CUL4B and DDB1 at the target gene promoters (Figure 3E). Knockdown of either CUL4B or DDB1 consistently led to a dramatic increase in pan‐H3 acetylation at the promoters of *E‐cadherin* and *AXIN2*; however, knockdown of either MTA1 or HDAC1 led to a limited reduction in the association of H2AK119ub1 with these promoters (Figure 3E), suggesting that CRL4B‐mediated H2AK119ub1 acts in conjunction with HDAC‐catalyzed pan‐H3 acetylation. In addition, analysis of two publicly available datasets (GSE27562 and GSE65194) revealed that the expression levels of E‐cadherin and AXIN2 were significantly negatively correlated with CUL4B expression, whereas MTA1 was significantly positively correlated with CUL4B expression in breast cancer samples (Figure S3B, Supporting Information 1). These results confirm that CUL4B and MTA1/NuRD are functionally associated through transcriptional repression of a cohort of target genes such as *E‐cadherin* and *AXIN2*.

### 2.4 CRL4B/NuRD(MTA1) Complex Promotes the Invasion of Breast Cancer Cells

**Figure 4:**
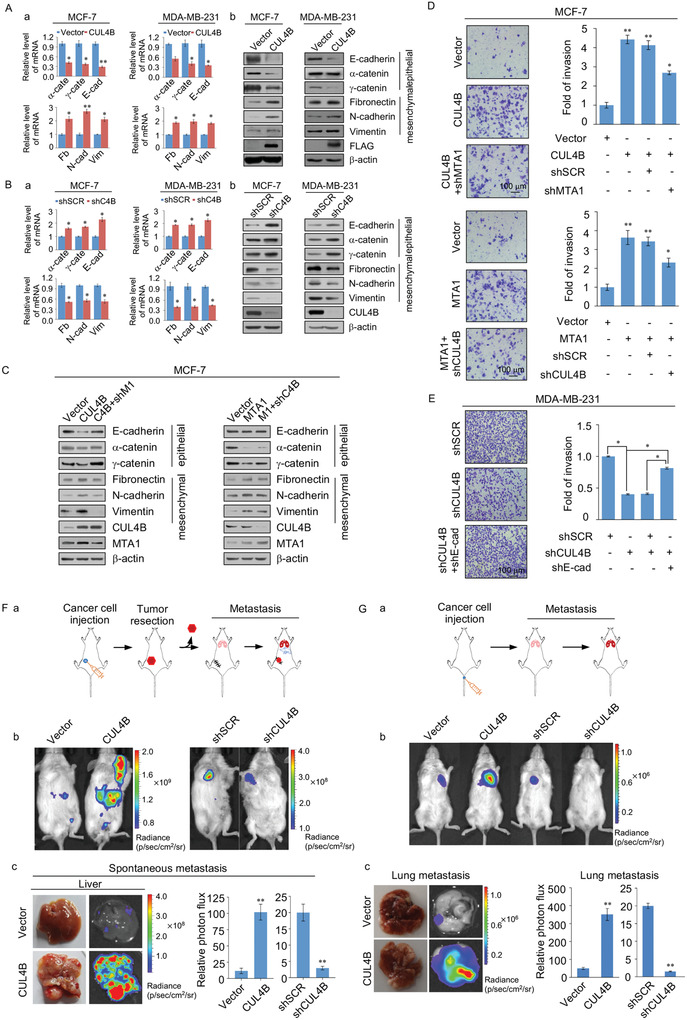
The CRL4B/NuRD (MTA1) complex promotes the invasion of breast cancer cells. A) RT‐PCR analysis of the mRNA expression patterns (a) of epithelial and mesenchymal markers and immunoblotting to assess the indicated protein levels (b) in MCF‐7 cells and MDA‐MB‐231 cells stably overexpressed CUL4B. C4B, CUL4B; *α*‐cate, *α*‐catenin; *γ*‐cate, *γ*‐catenin; E‐cad, E‐cadherin; Fb, Fibronectin; N‐cad, N‐cadherin; Vim, Vimentin. B) RT‐PCR analysis of the mRNA expression patterns (a) of epithelial and mesenchymal markers and immunoblotting to assess the indicated protein levels (b) in MCF‐7 cells and MDA‐MB‐231 cells stably decreased CUL4B. C4B, CUL4B; *α*‐cate, *α*‐catenin; *γ*‐cate, *γ*‐catenin; E‐cad, E‐cadherin; Fb, Fibronectin; Ncad, N‐cadherin; Vim, Vimentin. C) CUL4B or MTA1 was silenced in MCF‐7 cells stably transfected with vector, MTA1, or CUL4B, and the expression of epithelial and mesenchymal markers was assessed by western blotting. D) CUL4B or MTA1 were silenced in MCF‐7 cells stably transfected with vector, MTA1, or CUL4B. The cells were then prepared for invasion assays using Matrigel transwell filters. The invaded cells were stained and counted. The images represent one field under microscopy in each group. E) Transwell invasion assays were performed in MDA‐MB‐231 cells infected with lentiviruses carrying shCUL4B or in combination with lentiviruses carrying shE‐cadherin. E‐cad, E‐cadherin. F) The orthotopic mouse of spontaneous breast cancer metastasis experiments. a) Experimental design of the study. Mice were injected with the indicated MDA‐MB‐231‐Luc cells into the mammary fat pad and tumors were removed after reaching a volume of 300 mm^3^ (*n* = 5). b) Spontaneous metastases were quantified using bioluminescence imaging 3 weeks after removal of the primary tumors. c) Representative in vivo bioluminescent images of liver are shown (left panels). Metastatic photon flux was measured by bioluminescent measurement. G) MDA‐MB‐231‐Luc cells infected with lentiviruses carrying empty vector, CUL4B expression construct, control shRNA, or CUL4B shRNA were injected into the lateral tail veins of 6 week old female SCID mice (*n* = 6). a) Experimental design of the intravenously injected group. b) Lung metastasis was measured by quantitative bioluminescence imaging at 6 weeks after injection. c) The lung cancer specimens were quantified by in vitro bioluminescence imaging. A,B,D,E) Error bar represents mean ± SD of three independent experiments. *p < 0.05, **p < 0.01. Student's *t*‐test. F,G) Data are presented as mean ± SEM. *p < 0.05, **p < 0.01. Student's *t*‐test.

The CRL4B/NuRD(MTA1) complex was found to target several cellular signaling pathways critical for cell migration and invasion. In recent years, MTA1 was shown to be upregulated in several human cancers, particularly breast cancer.^[^ ^17^ ^]^ To explore the functional connection between the CRL4B and NuRD(MTA1) complexes, we investigated the role of the CRL4B/NuRD(MTA1) complex in the invasion and metastasis of breast cancer. To this end, changes in epithelial or mesenchymal markers were examined in CUL4B‐ and/or MTA1‐overexpressing MCF‐7 or MDA‐MB‐231 cells. CUL4B individually overexpression in MCF‐7 or in MDA‐MB‐231 cells led to a reduction in epithelial markers including *α*‐catenin, *γ*‐catenin, and E‐cadherin and induction of mesenchymal markers including fibronectin, N‐cadherin, vimentin at the mRNA and protein levels (**Figure** **4**A). Similarly, following individual knockdown of CUL4B in MCF‐7 or MDA‐MB‐231 cells, these EMT markers exhibited the opposite trend (Figure 4B). Consistently, overexpressing FLAG‐tagged CUL4B, both individually and in conjunction with MTA1, in nonmetastatic MCF‐7 cells led to reduced expression of epithelial markers as well as the induction of mesenchymal markers at the mRNA and protein levels (Figure S4A, Supporting Information 1). Individual or simultaneous knockdown of CUL4B and MTA1 in metastatic MDA‐MB‐231 cells resulted in increased expression of epithelial markers and reduced expression of mesenchymal markers at both the mRNA and protein levels (Figure S4B, Supporting Information 1).

In addition, knockdown of MTA1 in MCF‐7 cells stably expressing CUL4B or knockdown of CUL4B in MTA1‐expressing MCF‐7 cells, led to at least a partial elevation in the expression of epithelial markers and decrease in the expression of mesenchymal markers (Figure 4C); consistent with the phenotype observed through in vitro transwell invasion assays, the effect of CUL4B or MTA1 overexpression was diminished by knockdown of MTA1 or CUL4B in MCF‐7 cells (Figure 4D). These results indicate that CUL4B and MTA1 are functionally interdependent during EMT promotion. We then investigated the role of CUL4B in tumor invasion via in vitro CUL4B loss‐of‐function experiments using transwell invasion assays. CUL4B knockdown led to a threefold decrease in the cell invasion capability of MDA‐MB‐231 cells. In addition, E‐cadherin knockdown in CUL4B‐depleted MDA‐MB‐231 cells led to at least partial cell invasion (Figure 4E, Figure S4C, Supporting Information 1). These data indicate that CUL4B is important for breast cancer cell invasion by repressing EMT regulators.

We next investigated the possible role of CUL4B in breast cancer metastasis in vivo. MDA‐MB‐231 cells stably expressing firefly luciferase (MDA‐MB‐231‐Luc) were infected with lentiviruses carrying empty vector, CUL4B expression construct, control shRNA, or CUL4B shRNA. The indicated cells were then orthotopically implanted into 6 week old female NOD/SCID mice, and then the tumors were removed after reaching a volume of 300 mm^3^ (Figure 4F‐a).^[^ ^45^ ^]^ The results showed that overexpression of CUL4B could promote the tumor growth rate, while CUL4B knockout significantly reduced the tumor growth rate (Figure S4D, Supporting Information 1). Spontaneous metastases were verified by quantitative bioluminescence imaging 3 weeks after removal of the primary tumors. A metastatic event was defined as any detectable luciferase signal that was not located in the primary tumor. Compared to the control groups, we found that knockdown of CUL4B resulted in a significant decrease in tumor metastasis, while overexpression of CUL4B significantly promoted multiple organ metastasis (Figure 4F‐b,c, Figure S4E, Supporting Information 1). Thus, CUL4B could increase the early invasion ability of breast cancer cells. Moreover, similar results were also founded in the intravenously injected groups: compared to the control groups, knockdown of CUL4B led to significantly decreased lung metastases of MDA‐MB‐231‐Luc tumors, while dramatically increased lung metastases were observed in mice injected with MDA‐MB‐231‐Luc cells overexpressing CUL4B. Lung metastases were verified by bioluminescence imaging (Figure 4G). These results suggest that CUL4B significantly promotes the invasion and metastasis of breast cancer cells via coordination with the NuRD(MTA1) complex.

### 2.5 CRL4B/NuRD(MTA1) Complex is Recruited by the Transcription Factors Snail/ZEB2 to Promote Breast Cancer Metastasis

**Figure 5:**
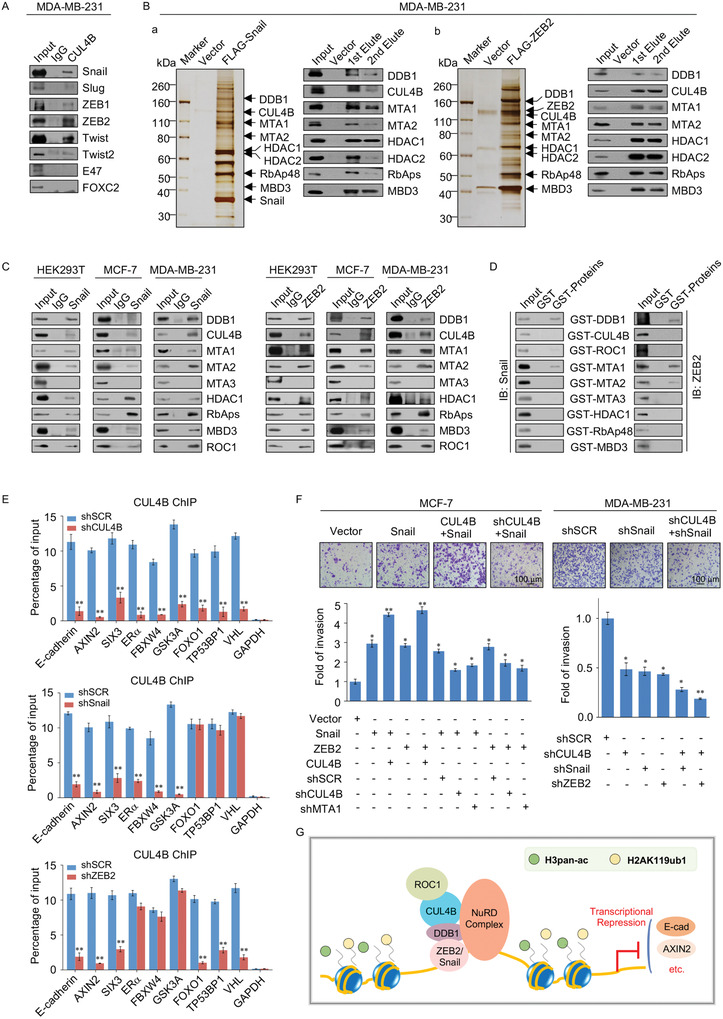
The CRL4B/NuRD(MTA1) complex was recruited by the transcription factors Snail/ZEB2 to promote breast cancer metastasis. A) Immunoprecipitation assays of MDA‐MB‐231 cells with anti‐CUL4B followed by immunoblotting with antibodies against the indicated proteins. B) Immunoaffinity purification and mass spectrometry analysis of Snail‐/ZEB2‐associated proteins in MDA‐MB‐231 cells (left panel). The column‐bound proteins were validated by western blotting using antibodies against the indicated proteins (right panel). C) Immunoprecipitation assays were performed with anti‐Snail or anti‐ZEB2 antibodies followed by immunoblotting with antibodies against the indicated proteins in HEK293T, MCF‐7, and MDA‐MB‐231 cells. D) GST pull‐down assays were performed with the indicated GST‐fused proteins and in vitro transcribed/translated Snail or ZEB2. E) qChIP experiments were performed in MDA‐MB‐231 cells transfected with shCUL4B, shSnail, shZEB2, or shSCR, using specific antibodies against CUL4B on representative target genes. GAPDH served as a negative control. F) Transwell invasion experiments were carried out in MCF‐7 cells and MDA‐MB‐231 cells; samples were transfected with the indicated specific shRNAs and/or expression constructs. The images represent one field under microscopy in each group. G) Schematic representation of the interaction between CRL4B and major EMT‐related transcription factors. E,F) Error bars represent mean ± SD of three independent experiments. *p < 0.05, **p < 0.01. Student's *t*‐test.

During the progression of EMT, transcription factors including Snail, Slug, ZEB1, ZEB2, Twist, Twist2, and E47 recruit various DNA/histone modification complexes to repress E‐cadherin and promote EMT.^[^ ^23^ ^]^ The coimmunoprecipitation assays showed that Snail, Slug, ZEB1, ZEB2, Twist, and Twist2 could interact with CUL4B, indicating that CUL4B is a key node for multiple tumor metastasis pathways and epigenetic regulatory networks (**Figure** **5**A). To better understand the mechanistic role between the CRL4B/NuRD(MTA1) complex and these transcription factors, we focused on Snail and ZEB2 in subsequent experiments, as they showed a clear interaction with CUL4B in the previous set of experiments. Affinity purification and silver staining were performed to identify Snail‐ or ZEB2‐interacting proteins in MDA‐MB‐231 cells (Figure 5B). DDB1, CUL4B, MTA1, MTA2, HDAC1, HDAC2, RbAp46/48, and MBD3 were detected in the anti‐FLAG‐Snail affinity‐purified eluate by western blotting analysis (Figure 5B‐a). Furthermore, the CRL4B/NuRD(MTA1) complex was copurified with ZEB2 (Figure 5B‐b). These results were confirmed by endogenous coimmunoprecipitation experiments with anti‐Snail or anti‐ZEB2 in HEK293T, MCF‐7, and MDA‐MB‐231 cells. All subunits of the CRL4B/NuRD(MTA1/2) complex showed efficient physical interactions with Snail and ZEB2; in contrast, MTA3 showed no association with neither Snail nor ZEB2, confirming our previous results (Figure 5C).^[^ ^20^ ^]^ Moreover, to investigate the molecular basis for the interaction between the CRL4B complex and Snail/ZEB2, GST pull‐down assays were performed using the indicated GST‐fused CRL4B/NuRD subunits and in vitro transcribed/translated Snail or ZEB2. Both DDB1 and MTA1/2 directly interacted with Snail and ZEB2, whereas MTA3 did not (Figure 5D). These results reveal that both Snail and ZEB2 are physically associated with CRL4B/NuRD(MTA1) by directly binding to DDB1 and MTA1/2. The detailed mass spectrometric results are shown in Tables S2 and S3 (Supporting Information 1).

To investigate whether CUL4B, Snail, and ZEB2 co‐occupied the target promoters, MDA‐MB‐231 cell clones with CUL4B, Snail, or ZEB2 stably depleted were generated by the indicated lentivirus‐delivered shRNA and shSCR. qChIP experiments were performed in the cells described above, using specific antibodies against CUL4B on representative target genes. The results indicated that depletion of either Snail or ZEB2 resulted in a marked reduction in the recruitments of CUL4B to the promoters of *E‐cadherin*, *AXIN2*, and *SIX3*. However, the binding of CUL4B to *ERα*, *FBXW4*, and *GSK3A* promoters were only affected by Snail, while the recruitments of *FOXO1*, *TP53BP1*, and *VHL* promoters by CUL4B were only affected by ZEB2. These results confirmed that CUL4B could be recruited by Snail and/or ZEB2 (Figure 5E).

Next, we explored the functional connection between Snail/ZEB2 and CRL4B. The results showed that individual and simultaneous overexpression of CUL4B and Snail in MCF‐7 cells resulted in reduced expression of epithelial protein markers as well as the increased expression of mesenchymal protein markers (Figure S5A, left panels, Supporting Information 1). Individual or simultaneous knockdown of CUL4B and Snail in MDA‐MB‐231 cells resulted in the opposite trend (Figure S5A, right panels, Supporting Information 1). Moreover, individual or simultaneous knockdown or overexpression of CUL4B and ZEB2 showed similar results (Figure S5B, Supporting Information 1). To further confirm this functional link, we investigated the influence of CUL4B, MTA1, Snail, and ZEB2 on the cellular behavior of breast cancer cells in vitro using transwell invasion assays. Overexpression of Snail or ZEB2 in MCF‐7 cells resulted in an elevated cell invasion potential, whereas knockdown of Snail or ZEB2 led to a decrease in the cell invasion potential of MDA‐MB‐231 cells. Moreover, CUL4B further enhanced the Snail‐ and ZEB2‐ induced invasion capacity of MCF‐7 cells; similarly, CUL4B knockdown diminished the effect of Snail or ZEB2 knockdown on the invasion potential of MDA‐MB‐231 cells (Figure 5F). In contrast, the effect of individual Snail or ZEB2 overexpression on increasing the invasion potential of MCF‐7 cells was partially counterbalanced by knockdown of CUL4B or MTA1 using lentivirus‐delivered shRNA (Figure 5F, left panels). These results indicate a critical role for the Snail/NuRD(MTA1)/CRL4B and ZEB2/NuRD(MTA1)/CRL4B complexes in regulating EMT and cell invasion (Figure 5G), suggesting that the CRL4B/NuRD(MTA1) complex is a molecular hub controlling EMT‐related epigenetic regulation networks.

### 2.6 CUL4B Depletion Abolished Hypoxia/Wnt/Notch‐Induced EMT, and CUL4B is Negatively Regulated by the Estrogen‐ER*α*‐GATA3 Axis

In cancer metastasis, several signaling pathways, including hypoxia, Wnt, Notch, Hedgehog, and TGF*β*, play vital roles in EMT.^[^ ^46^, ^47^, ^48^, ^49^, ^50^ ^]^ Therefore, we explored whether CUL4B is functionally associated with these breast cancer EMT‐associated signaling pathways, which were also enriched in our CUL4B ChIP‐seq experiments. MCF‐7 cells were treated with CoCl_2_, recombinant human Wnt‐3a (WNT3A), and recombinant human Jagged‐1 (JAG1) to stimulate hypoxia, Wnt, and Notch pathways, respectively.^[^ ^51^, ^52^ ^]^

**Figure 6:**
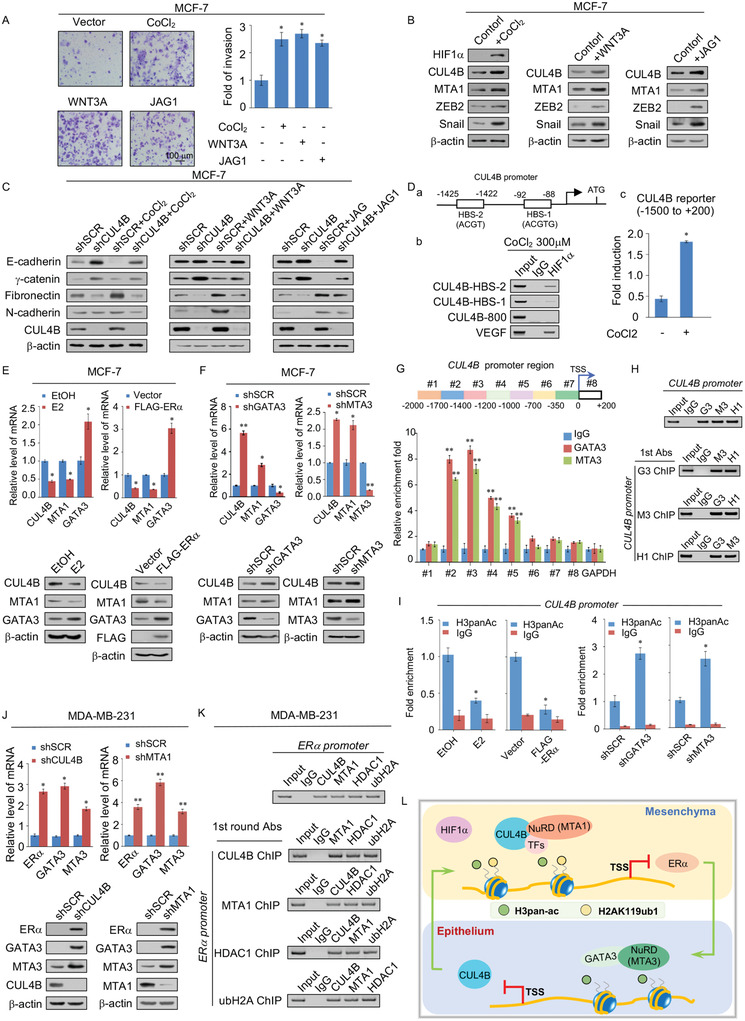
CUL4B depletion abolishes hypoxia/Wnt/Notch‐induced EMT and CUL4B is negatively regulated by the estrogen‐ER*α*‐GATA3 Axis. A) Transwell invasion experiments were carried out in MCF‐7 cells treated with CoCl_2_, WNT3A, or JAG1. The images represent one field under microscopy in each group. B) MCF‐7 cells were treated with CoCl_2_, WNT3A, and JAG1 to stimulate the hypoxia, Wnt, and Notch signaling pathways, respectively. Expression levels of CUL4B, MTA1, Snail, and ZEB2 were then analyzed by western blotting. C) MCF‐7 cells were transfected with the indicated specific lentivirus‐delivered shSCR or shCUL4B, and simultaneously treated with or without CoCl_2_, WNT3A, and JAG1 to stimulate the hypoxia, Wnt, and Notch signaling pathways, respectively. The expression of epithelial and mesenchymal markers was measured by western blotting. D) Schematic representation of the CUL4B promoter region (a). ChIP assays in CoCl_2_ treated MCF‐7 cells with antibodies against HIF1*α* on the predicted sites of the CUL4B gene promoter (b). A CUL4B‐luciferase reporter was constructed with the proximal promoter region (‐1500 to +200 to TSS) using the pGL4‐CMV‐reporter vector. MCF‐7 cells transfected with CUL4B‐luciferase reporters, together with renilla reporters as an internal control, were exposed to CoCl_2_ (c). E) The expression of CUL4B, MTA1, and GATA3 was detected by qRT‐PCR (upper panels) and western blotting (lower panels) in MCF‐7 cells starved for 3 days and treated with 17*β*‐estradiol (E2) for 24 h or in MCF‐7 cells transfected with FLAG‐Vector or FLAG‐ER*α* construct. F) Knockdown of GATA3 or MTA3 in MCF‐7 cells to measure CUL4B and MTA1 expression at both the mRNA (upper panels) and protein levels (lower panels). G) Primer pairs including #1 to #8 were synthesized to cover the promoter region of *CUL4B* as indicated. qChIP‐based promoter‐walk experiments were performed using MCF‐7 cells, and the enrichment of GATA3 and MTA3 were mapped to four regions of the *CUL4B* promoter. H) ChIP and Re‐ChIP experiments were performed in MCF‐7 cells with the indicated antibodies against GATA3, MTA3, and HDAC1 at the *CUL4B* promoter. G3, GATA3; M3, MTA3; H1, HDAC1. I) MCF‐7 cells were treated with or without E2, or transfected with vector or overexpression ER*α*, or infected with shSCR, shGATA3, shMTA3. qChIP analysis of the recruitment of the *CUL4B* promoters was performed using the H3panAc antibodies. Purified rabbit IgG was used as a negative control. J) MDA‐MB‐231 cells were transfected with the indicated specific lentivirus‐delivered shCUL4B or shMTA1. The mRNA and protein expression levels of ER*α*, GATA3, and MTA3 were measured by RT‐PCR (upper panels) and western blotting (lower panels), respectively. K) ChIP and Re‐ChIP experiments were performed in MDA‐MB‐231 cells with the indicated antibodies against CUL4B, MTA1, HDAC1 and ubH2A at the *ER* *α* promoter. ubH2A, H2AK119ub1. L) Schematic representation showing the proposed reciprocal feedback regulatory mechanism model for the CUL4B‐dependent EMT epigenetic regulation network and the ER*α*‐GATA3‐CUL4B/MTA1 axis in breast cancer carcinogenesis. A,D–G,I,J) Error bars represent mean ± SD of three independent experiments. *p < 0.05, **p < 0.01. Student's *t*‐test.

As shown in **Figure** **6**A, the invasive potential of nonmetastatic MCF‐7 cells increased after CoCl_2_, WNT3A, and JAG1 treatment, indicating effective pathway induction. CUL4B, MTA1, ZEB2, and Snail expression was significantly increased after stimulation with CoCl_2_, WNT3A, and JAG1, indicating that these factors were responsive to the agonists of these three pathways, as expected. This suggests that CUL4B, MTA1, ZEB2, and Snail participate in these critical signaling pathways (Figure 6B). Moreover, western blotting results indicated that depression of epithelial markers (E‐cadherin, and *γ*‐catenin) or induction of mesenchymal markers (N‐cadherin and vimentin) associated with activation of the hypoxia, Wnt, or Notch signaling pathways can be dramatically offset by CUL4B knockdown (Figure 6C). This suggests that CUL4B is a hub factor for EMT‐promoting signaling pathways. In addition, bioinformatics analysis of the *CUL4B* promoter using BIOBASE revealed multiple potential HIF‐1 binding sequences (Figure 6D‐a).^[^ ^53^ ^]^ ChIP assays identified that in anoxic MCF‐7 cells, HIF1*α* occupies the *CUL4B* promoter mainly at the HIF‐1 binding site 2 (ACGT) regions. Moreover, the fluorescence intensity of the CUL4B luciferase reporter containing the CUL4B promoter (‐1500 to +200) was significantly increased in MCF‐7 cells after simulated CoCl_2_ treatment which could mimic hypoxia condition by HIF1*α* upregulation (Figure 6D‐b,c). Thus, HIF1*α* can directly activate CUL4B expression by binding to the hypoxia hormone response element (HRE) on the *CUL4B* promoter in breast cancer cells. These results suggest a critical role for CUL4B in the hypoxia, Wnt, and Notch pathways, and that CUL4B is a key downstream effector of many EMT‐promoting signaling pathways.

Our results suggested that CUL4B and ER*α* are functionally associated. To address this prediction, MCF‐7 cells were cultured in steroid‐depleted and phenol red‐free medium for 3 days and treated with 17*β*‐estradiol (E2). We then investigated the expression of CUL4B and MTA1 in these cells by qRT‐PCR and western blotting. As expected, both the mRNA and protein levels of CUL4B and MTA1 were markedly decreased after E2 treatment (Figure 6E, left panels). Overexpression of FLAG‐ER*α* also led to the decreased expression of CUL4B and MTA1 at both the mRNA and protein levels in MCF‐7 cells (Figure 6E, right panels). Expression of the well‐known ER*α*‐activated gene *GATA3*, which is important for maintaining a stable epithelial morphology,^[^ ^54^ ^]^ was increased under both E2 stimulation and ER*α* overexpression, as reported previously (Figure 6E). However, because the classical function of E2 is to stimulate ER*α* through transcriptional activation, the negative regulation of CUL4B expression by ER*α* may be controlled by the GATA3/NuRD (MTA3) complex, as seen in our previous work.^[^ ^20^ ^]^ In agreement with this, knockdown of GATA3 resulted in increased expression of CUL4B and MTA1 at both the mRNA and protein levels in MCF‐7 cells (Figure 6F, left panels). Similarly, knockdown of MTA3 elevated the expression of both CUL4B and MTA1 (Figure 6F, right panels). These results agree with those of our earlier study showing that GATA3 selectively recruited the NuRD(MTA3) complex for transcriptional repression and metastasis suppression.^[^ ^20^ ^]^

According to previous reports, both GATA3 and MTA3 are the transcriptional activation target genes of ER*α*.^[18,54]^ For E2 activated ER*α* is mainly responsible for transcriptional activation,^[^ ^55^ ^]^ we propose that ER*α* could repress CUL4B through the induction of GATA3 and MTA3. To gain a deeper insight into the mechanism through which the GATA3/NuRD(MTA3) complex regulates CUL4B transcription, qChIP‐based promoter‐walk experiments were performed using MCF‐7 cells. The results showed that the significant enrichments of GATA3 were mapped to four regions of the *CUL4B* promoter at upstream ∼1700 to ∼700 promoter regions. Moreover, MTA3 is also bound to the similar regions (Figure 6G). To further substantiate the hypothesis that ER*α*‐GATA3/NuRD (MTA3) negatively regulates CUL4B, sequential ChIP or ChIP/Re‐ChIP experiments using specific antibodies against GATA3, MTA3, and HDAC1were performed in MCF‐7 cells. The results showed that the *CUL4B* promoter was immunoprecipitated with antibodies against GATA3 from the first‐round ChIP and could then be reimmunoprecipitated with antibodies against MTA3 and HDAC1, as indicated. Similar results were obtained when the initial ChIP was performed with antibodies against MTA3 and HDAC1 (Figure 6H). These data confirmed that the GATA3/(MTA3)NuRD complex occupied the *CUL4B* promoter as one protein complex. In addition, the results of qChIP experiments showed that the levels of H3panAc decreased in the CUL4B promoter with E2 treatment or ER*α* overexpression while increased in GATA3 or MTA3 depleted MCF‐7 cells (Figure 6I). Furthermore, qChIP and ChIP assays for the *CUL4B*, *MTA1*, and *ZEB2* promoters in MCF‐7 cells indicated that GATA3 and MTA3 bind to the promoters of all three genes (Figure S6A, Supporting Information 1). Taken together, these results suggested that the GATA3/NuRD (MTA3) complex could inhibit CUL4B transcription by deacetylation of the *CUL4B* promoter. On the other side, CUL4B and MTA1 knockdown in MDA‐MB‐231 cells resulted in increased expression of ER*α*, GATA3, and MTA3 at the mRNA and protein levels (Figure 6J). ChIP and Re‐ChIP assays were performed on the promoters of *ERα* (Figure 6K). The results showed that the CUL4B/NuRD(MTA1) complex could directly bind to the *ERα* promoter. Moreover, qChIP assays showed that suppression of CUL4B resulted in a significant reduction in the recruitment of MTA1 and HDAC1 to the *ERα* promoter, accompanied by a decrease in H2AK119ub1 level and an increase in H3panAc level (Figure S6B, Supporting Information 1). In addition, analysis of three public clinical datasets (GEO: GSE65194, GSE5460, GSE19615) showed an obvious positive correlation between the expression of CUL4B and HIF1*α*, a significant negative correlation between ER*α* and CUL4B and between GATA3 and CUL4B expression, and a significant positive correlation between ER*α* and GATA3 expression (Figure S6C, Supporting Information 1). Collectively, these results suggest that CRL4B is a key node within the EMT epigenetic regulation network which is positively regulated by EMT‐promoting pathways while negatively regulated by the estrogen signaling pathway, and a reciprocal feedback regulatory loop exists between CRL4B/NuRD(MTA1) and the E2/ER*α*‐GATA3/NuRD(MTA3) axis in controlling EMT and progression of breast cancer (Figure 6L).

### 2.7 CUL4B Promotes the Growth of Breast Tumor Xenografts in NOD/SCID Mice by Upregulating the Breast Cancer Stem Cell Population

**Figure 7:**
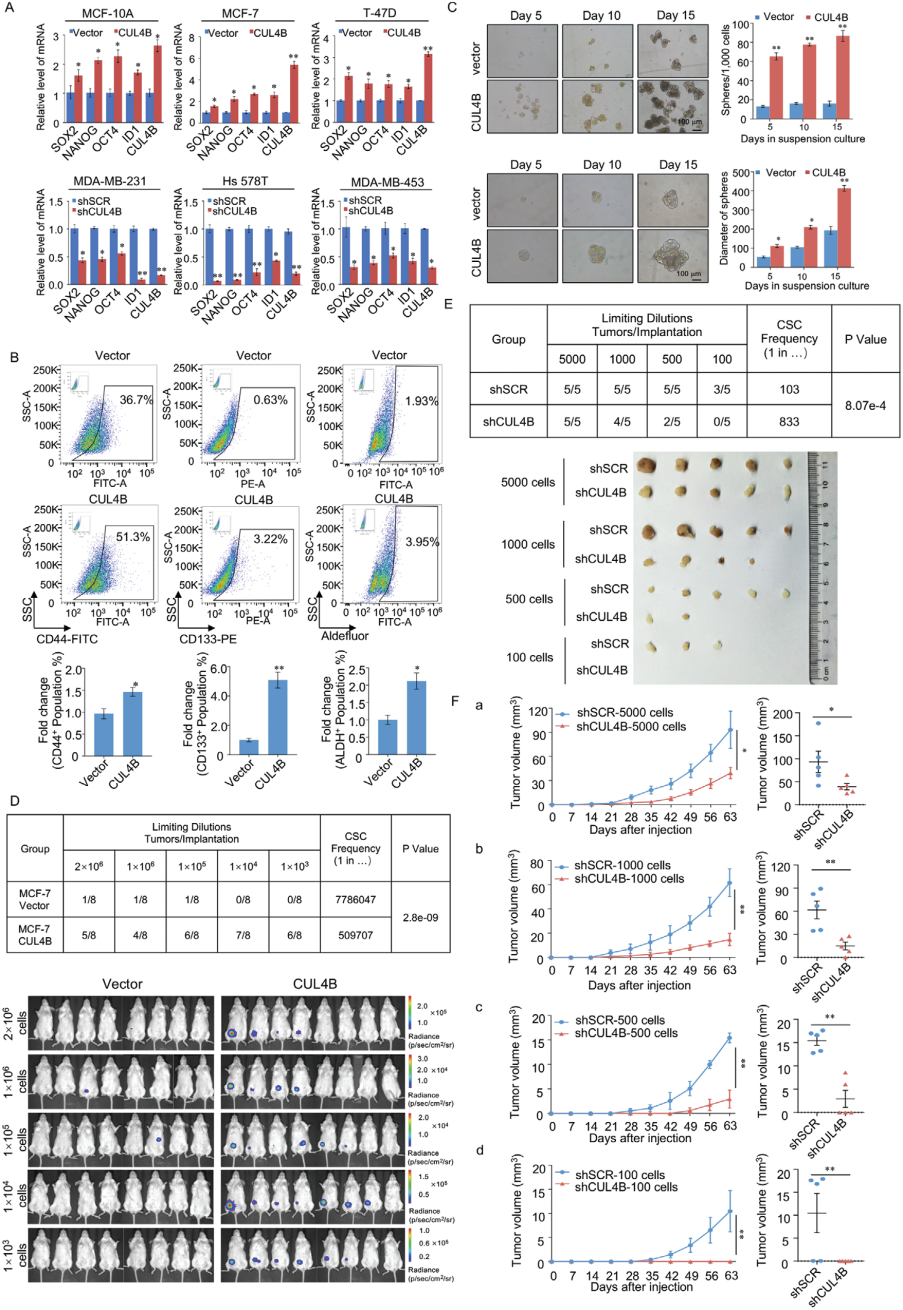
CUL4B promotes the growth of breast tumor xenografts in NOD/SCID mice by upregulating the breast cancer stem cell population. A) RT‐PCR data for the relative mRNA expression levels of *NANOG*, *SOX2*, *OCT4*, and *ID1* in CUL4Boverexpressing MCF‐10A, MCF‐7, or T‐47D cells, as well as in CUL4B‐knockdown MDA‐MB‐231, Hs 578T, or MDA‐MB‐453 cells. B) Stable CUL4B‐overexpressing cell line was established with MCF‐7 cells. These cells were analyzed for CD44, CD133, and ALDH by using flow cytometry. C) MCF‐7 cells were transfected with an empty vector or CUL4B expression construct. Representative images of the indicated mammospheres grown in suspension culture for 15 days. Mammospheres were generated from single‐cell cultures of the vector, and CUL4B was imaged on the indicated day of suspension culture. A–C) Error bars represent mean ± SD of three independent experiments. *p < 0.05, **p < 0.01. Student's *t*‐test. D) Tumor incidence of CUL4B‐expressing MCF‐7 cells after being injected into the mammary gland fat pads of NOD/SCID mice at various dilutions (*n* = 8 in each group). The frequency of CSCs was calculated using the Extreme Limiting Dilution Analysis (ELDA) software (http://bioinf.wehi.edu.au/software/elda/index.html). E) Tumorigenicity was determined by injecting MDA‐MB‐231‐shSCR or MDA‐MB‐231‐shCUL4B cells at various dilutions (*n* = 5 in each group). The stem cell frequency in xenograft tumors was calculated by the ELDA. F) Tumor growth curve of different numbers (5000, 1000, 500, and 100) of MDA‐MB‐231‐shSCR or MDA‐MB‐231‐shCUL4B cells were monitored over the indicated time period. Tumor volumes at the endpoint were shown in the right panels. Data are presented as mean±SEM. *p < 0.05, **p < 0.01. Student's *t*‐test.

Based on our observations that CUL4B was positively correlated with tumor malignancy, we investigated whether CUL4B affects stem‐like phenotypes in normal and breast cancer cells. The stem cell markers Nanog, Sox2, Oct4, and ID1 were upregulated in non‐invasive MCF‐10A, MCF‐7, and T‐47D cells stably expressing CUL4B (**Figure** **7**A, upper panels). However, their expression declined in response to CUL4B knockdown in invasive MDA‐MB‐231, Hs 578T, and MDA‐MB‐453 cells (Figure 7A, lower panels). CD44^+^/CD24^–/low^ has been reported as a marker for breast cancer stem cells.^[^ ^26^ ^]^ MCF‐10A, MCF‐7, and T‐47D cells stably expressing CUL4B showed significantly elevated CD44 expression and significantly reduced CD24 expression (Figure S7A, Supporting Information 1). In contrast, CD44 expression was markedly reduced, whereas CD24 expression was induced in CUL4B‐knockdown MDA‐MB‐231, Hs 578T, and MDA‐MB‐453 cells (Figure S7B, Supporting Information 1). Moreover, fluorescence‐activated cell sorting (FACS) analysis results showed that the populations of cells positive for the biomarkers of cancer stem cells, CD44, CD133, and ALDH, were significantly increased among cells overexpressing CUL4B (Figure 7B). These results demonstrate that CUL4B is essential for the development of cancer stemness.

To further elucidate whether CUL4B promotes MCF‐7 cells to develop into CSCs and repopulate from single cells, we analyzed the effect of CUL4B on mammosphere formation.^[^ ^56^ ^]^ Analysis from days 5 to 15 of treatment indicated that CUL4B effectively promoted the growth of MCF‐7‐derived mammospheres in terms of both size and cell number (Figure 7C). Next, to assess the effect of CUL4B on the breast CSC population, MCF‐7 cells engineered to stably express firefly luciferase lentiviruses carrying an empty vector or CUL4B were subcutaneously injected into the mammary fat pads of NOD/SCID mice at limit dilutions (2 × 10^6^, 1 × 10^6^, 1 × 10^5^, 1 × 10^4^, and 1 × 10^3^) to determine their tumor formation ability. We did not implant any E2 pills, and the growth of implanted cells was visualized via bioluminescence at 4 weeks after injection. Unexpectedly, although most control MCF‐7 cells did not form tumors without E2 stimulation, tumors formed in mice injected with as few as 1 × 10^3^ luciferase‐labeled MCF‐7‐CUL4B cells (6/8); CUL4B‐overexpressing cells showed a significant increase in CSC frequency and higher tumor‐formation capacity than those in the control group (Figure 7D, Figure S7C, Supporting Information 1). Furthermore, different numbers (5000, 1000, 500, and 100) of MDA‐MB‐231‐shSCR or MDA‐MB‐231‐shCUL4B cells were injected into the mammary fat pads of NOD‐SCID female mice, and tumor growth was monitored for approximately 2 months. The results showed that mice in the shSCR group developed tumors with as few as 100 cells, whereas those in the shCUL4B group did not develop any tumors (Figure 7E), suggesting that the possibility of tumor formation was greatly reduced after CUL4B depletion. Furthermore, the tumor growth rate was also significantly suppressed in the shCUL4B groups (Figure 7F), similar to our previous report.^[^ ^5^ ^]^ These data suggest that CUL4B dramatically induces stemness in breast cancer cells.

### 2.8 CUL4B is Upregulated in Breast Cancer and is a Potential Cancer Biomarker

**Figure 8:**
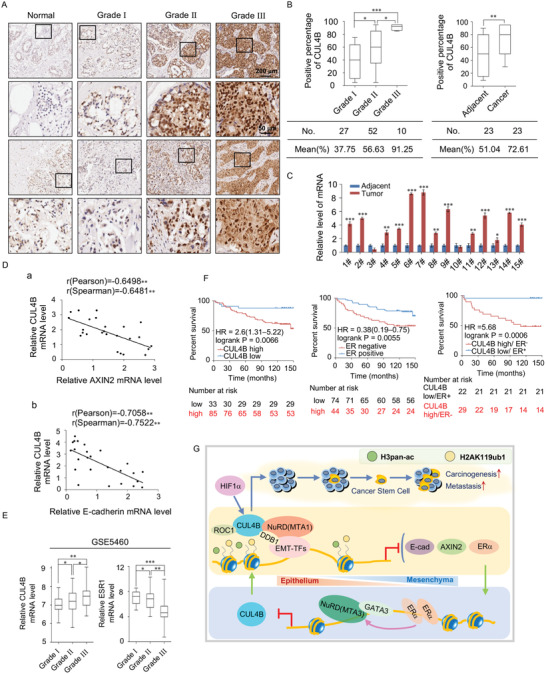
CUL4B is upregulated in breast cancer and is a potential cancer biomarker. A) Immunohistochemical staining of CUL4B in normal breast tissue and breast tumors (histological grades I, II, & III). For each grade, representative photos of two specimens are shown. B) The positively stained nuclei (in percentages) in grouped samples, as well as 23 paired samples were analyzed by a two‐tailed unpaired *t*‐test. C) The expression of *CUL4B* mRNA was upregulated in breast carcinoma samples. Total RNA in paired samples of 15 breast carcinoma tissues paired with adjacent normal mammary tissues was extracted and analyzed for CUL4B expression via qRT‐PCR. D) Analysis of *CUL4B*, *E‐cadherin*, and *AXIN2* expression by qRT‐PCR in 25 breast carcinoma samples. The relative level of CUL4B expression was plotted against that of E‐cadherin or AXIN2. E) Bioinformatics analysis of a GEO public dataset (GEO: GES5460) for the expression of CUL4B and ER*α* in grade I–III breast carcinoma samples. F) Kaplan‐Meier survival analysis of clinical data for the relationship between survival time and CUL4B/ER*α* expression signatures in breast cancer. Survival curves were calculated using Kaplan–Meier method. Log‐rank tests were used for the statistical analysis. G) The proposed regulatory mechanisms of the CRL4B/NuRD(MTA1) complex in breast carcinogenesis. E‐cad, E‐cadherin. B–E) Data are presented as mean ± SEM. *p < 0.05, **p < 0.01, ***p < 0.001. Student's *t*‐test.

To confirm the role of CUL4B in tumorigenesis, we collected 125 breast carcinoma samples from patients with breast cancer and performed tissue microarrays by immunohistochemical staining to examine the expression of CUL4B (**Figure** **8**A). CUL4B was significantly upregulated in tumors, and CUL4B expression levels were positively correlated with tumor histological grades (Figure 8B, left panel). In addition, the results revealed a remarkably significant increase in CUL4B expression in tumors compared to in adjacent normal breast tissue (Figure 8B, right panel). Moreover, in 14 of 15 selected paired samples of each tumor grade, the mRNA expression of *CUL4B* was upregulated in tumor tissues compared to in adjacent tissues (Figure 8C). Statistical analysis revealed significant negative correlations with a Pearson correlation coefficient of ‐0.7058 and ‐0.6498 when the relative expression of E‐cadherin and AXIN2, respectively, were plotted against that of CUL4B in 25 samples (Figure 8D). Strikingly, analysis of a public dataset (GEO: GSE5460) supported that the expression of CUL4B is positively correlated with the histological grades of tumors; in contrast, ER*α* expression showed the opposite trend (Figure 8E). In addition, analysis of METABRIC public datasets,^[^ ^57^ ^]^ obtained from cBioPortal,^[^ ^57^, ^58^ ^]^ indicated that CUL4B expression levels were positively correlated with several molecular subtypes of breast cancer, including the Luminal, Her2, and Basal subtypes, while ER*α* expression showed the opposite trend (Figure S8A, Supporting Information 1).

Finally, to determine the clinicopathological relevance of our results, we analyzed the expression of CUL4B and its correlation with the clinical behaviors of patients with breast cancer. Kaplan‐Meier survival analysis of CUL4B performed using an online database (http://kmplot.com/analysis/) revealed that increased expression of CUL4B, HIF1*α*, Snail, and ZEB2 were significantly positively associated with a poor overall survival rate in patients with breast cancer (Figure S8B, left and middle panels, Supporting Information 1). In contrast, increased ER*α* and GATA3 expression were associated with a higher overall survival rate in patients with breast cancer (Figure S8B, right panels, Supporting Information 1). A Kaplan‐Meier survival plot of CUL4B and ER*α* using our clinical data showed similar results (Figure 8F, left and middle panels). Further stratification of patient groups based on the inverse expression of ER*α*/CUL4B improved the predictive ability of CUL4B (Figure 8F, right panel), supporting CUL4B as a significant predictor of survival and a potential diagnostic marker. The proposed regulatory mechanisms of the CRL4B/NuRD(MTA1) complex in controlling EMT and stem cell properties of breast carcinogenesis are described in Figure 8G.

## 3 Discussion

In this study, we demonstrated that CUL4B transcriptional activity is associated with HDAC activity, as CRL4B interacted—both physically and functionally—with the four class I HDAC multiprotein corepressor complexes MTA1/NuRD, SIN3A, REST/CoREST, and NCoR/SMRT. Previously, we revealed that CRL4B is a transcriptional repressor and that the transcription repressive function of CUL4B is linked by catalyzing H2AK119 monoubiquitination and coordinating PRC2‐catalyzed H3K27me3.^[^ ^5^ ^]^ The CRL4B/PRC2 complex epigenetically regulates somatostatin secretion and facilitates glucose homeostasis in pancreatic islets.^[^ ^59^ ^]^ Subsequent research showed that CRL4B facilitates H3K9 tri‐methylation and DNA methylation through catalyzing H2AK119 mono‐ubiquitination.^[^ ^6^ ^]^ Herein, our identification of the physical association and functional link of CUL4B with four HDAC multiprotein corepressor complexes expanded the enzymatic repertoire of the deacetylase activity of CUL4B, providing a molecular basis for the interplay between histone ubiquitination and deacetylation in chromatin remodeling.

Previous studies showed that CRL4B is a histone modification enzyme for H2AK119ub1, which is the molecular basis for the interplay between histone ubiquitination/methylation and DNA methylation in chromatin remodeling.^[^ ^5^, ^6^ ^]^ We uncovered that CRL4B‐modified H2K119ub1 synergizes with histone deacetylation, expanding the enzymatic repertoire of the CUL4B to the deacetylase activity. HDAC inhibitors are hotspots for tumor‐targeted therapy and are first‐generation epigenetic modulators approved for clinical use, such as in myelodysplastic syndrome therapy. Moreover, the HDAC inhibitor SAHA was found to synergize with JQ1 to suppress advanced pancreatic ductal adenocarcinoma effectively.^[^ ^60^ ^]^ In addition, HDAC inhibitors have broad application prospects in combination with checkpoint inhibitors due to the ability of HDAC inhibitors to modulate the transcriptome of cancer and immune cells.^[^ ^61^ ^]^ Therefore, CUL4B inhibitors are potential epigenetic therapy drugs.

The physical association of CUL4B with multiple transcription corepressor complexes was an unexpected and confusing result. Thus, how the functional networks of the multiprotein complexes cooperate through physical interactions as well as the biological significance of this must be elucidated. A possible explanation is that CUL4B interacts with a special corepressor complex in a certain cellular microenvironment. In the present study, we demonstrated that CUL4B regulates extensive gene networks in breast cancer cells through interactions with multiple corepressor complexes; *E‐cadherin*, *AXIN2*, and *ERα* were regulated by the CRL4B/NuRD(MTA1) complex; *ATM, p21*, and *p18* by the CRL4B/SIN3A complex; *BDNF, LAMA5*, and *ROBO3* by the CRL4B/CoREST complex; and *AKT1, TRAF4*, and *TSC1* by the CRL4B/NCoR/HDAC3 complex. These genes were known to play critical roles in EMT, cell cycle progression, and axon guidance.^[^ ^62^, ^63^, ^64^ ^]^ Our findings demonstrated that CRL4B and the NuRD(MTA1) complex were physically associated and functionally linked to the promotion of EMT progression and the invasion of breast cancer cells. Additionally, we identified a molecular connection between CRL4B and the NuRD(MTA1) complex in EMT progression, as CUL4B and MTA1 were found to be functionally complementary. MTA1, MTA2, and MTA3 are thought to form different NuRD complexes as characteristic subunits. MTA1 and MTA2 have emerged as one of the most upregulated genes in several human cancers, including breast and endometrial cancer, whereas MTA3 may be downregulated.^[^ ^65^ ^]^ CRL4B directly interacted with MTA1/2 instead of MTA3, indicating that CRL4B is selective for the subunits of the NuRD complex. Previously, we showed that GATA3 and SIX3 specifically recognize and recruit the NuRD(MTA3) complex.^[^ ^20^, ^66^ ^]^ In the current study, we found that CRL4B, as a histone ubiquitinating enzyme, also has a preference for the NuRD subunit and interacted with NuRD(MTA1).

We further showed that the CRL4B/NuRD(MTA1) complex is recruited by a series of transcription factors, especially the representative transcription factors, Snail and ZEB2, two critical regulators of EMT.^[^ ^25^ ^]^ Snail is a C2H2 zinc‐finger protein, which has been implicated in various processes relating to cell differentiation and survival. Snail confers migratory and invasive properties to epithelial cells through repression of epithelial markers like E‐cadherin and claudins.^[^ ^67^ ^]^ Meanwhile, ZEB2 is an essential regulator of EMT and MET processes during development and tumorigenesis by forming a double‐negative feedback loop with several microRNA species, especially the miR‐200 family.^[^ ^68^ ^]^ Both Snail and ZEB2 are involved in cellular signaling pathways (e.g., hypoxia and Wnt pathways) promoting tumorigenesis.^[^ ^47^, ^69^ ^]^ Notably, CUL4B is also functionally linked to a number of pathways associated with tumor progression, such as the hypoxia, Wnt, and Notch pathway, which play essential roles in promoting EMT.^[^ ^48^ ^]^ Hypoxia is an important feature in hematologic tumors and solid tumors and the hypoxia pathway can regulate the cell cycle, prevent apoptosis, and maintain stem cell properties.^[^ ^70^ ^]^ The Wnt signal transduction cascade is a major regulator of development, and mutations of the Wnt signaling pathway components are responsible for a variety of cancers; for example, APC, a negative regulatory complex component of Wnt, is mutated in colon cancer, resulting in activation of the Wnt pathway.^[^ ^71^ ^]^ The Notch signaling pathway is essentially a hallmark of cancer, and the gain‐of‐function or loss‐of‐function mutations of Notch have been implicated in cancers. Moreover, Notch is involved in cancer cell metabolism, survival, and drug resistance as well as maintenance of cancer stem cells, EMT, and genomic instability.^[^ ^72^ ^]^

It is worth noting that CUL4B is positively regulated and transactivated by HIF1*α* directly. MTA1 upregulates and stabilizes HIF1*α* under hypoxic conditions via the MTA1/HDAC1 complex, which also stimulates HIF1*α* transcription, promoting angiogenesis.^[^ ^44^ ^]^ HDAC3, another protein that physically associates with CUL4B, was shown to be essential for hypoxia‐induced EMT and metastatic phenotypes.^[^ ^73^ ^]^ However, the role of CUL4B in other pathways is currently little known. As reported previously, CUL4B activates Wnt/*β*‐catenin signaling by stabilizing *β*‐catenin in response to GSK3‐mediated degradation in hepatocellular carcinomas.^[^ ^74^ ^]^ Despite this, the mechanism behind these phenotypes requires further investigation. Although the classical response elements of the Wnt or Notch pathways were not directly predicted on the *CUL4B* promoter, we found that the expression levels of CUL4B, MTA1, Snail, and ZEB2 increased during pathway activation, suggesting that signaling pathways can promote EMT and tumor metastasis through this regulatory network. CUL4B knockdown during signaling pathway activation directly leads to complete collapse of the network function and the signal cannot be transmitted, suggesting that CUL4B is a key and necessary factor in these pathways that play essential roles in promoting tumor metastasis. The interaction of CUL4B with the Wnt and Notch signaling pathways, as well as its contribution to breast cancer progression, remains to be fully elucidated; thus, further research is necessary.

Patients with ER*α* ^–^ breast cancers are normally associated with shorter survival times.^[^ ^75^ ^]^ GATA3 is strongly correlated with ER*α* expression and both are involved in a cross‐regulatory feedback loop.^[^ ^54^, ^76^ ^]^ In this study, we found that CUL4B/NuRD(MTA1) can directly inhibit ER*α*. Conversely, CUL4B and MTA1 levels markedly decreased after E2 stimulation and ER*α* overexpression. Furthermore, GATA3 and MTA3 transcriptionally repressed the expression of CUL4B, MTA1, and ZEB2. In return, CUL4B and MTA1 depletion resulted in increased expression of ER*α*, GATA3, and MTA3. Moreover, Snail is directly transcriptional repressed by MTA3^[^ ^18^ ^]^ and ZEB2 represses MTA3 expression through the formation of a ZEB2/G9A/NuRD(MTA1) complex.^[^ ^20^ ^]^ Evidently, a reciprocal feedback regulatory loop exists during breast cancer progression in which loss‐of‐function of ER*α* and GATA3 leads to elevated expression of CUL4B and MTA1. We also found that CUL4B can be recruited by Snail and ZEB2, cooperate with the NuRD(MTA1) complex, and inhibit the expression of ER*α* through transcriptional regulation, thus further reducing the intracellular expression levels of ER*α* and GATA3/MTA3. The CRL4B/NuRD(MTA1) complex could also promote cell response to the EMT and CSC‐related pathways, such as hypoxia, Wnt, and Notch, thereby promoting tumor metastasis as well as acquisition and maintenance of stem cell‐like properties.

The biological effects elicited by the EMT program are not only existed in cancer metastasis, as the EMT of human mammary epithelial cells can produce cells with properties associated with mammary epithelial stem cells, including the expression of stem cell markers and an increased ability to form mammospheres.^[^ ^33^, ^77^ ^]^ Moreover, several signaling pathways link EMT to self‐renewal and tumor formation.^[^ ^78^, ^79^, ^80^, ^81^, ^82^ ^]^ Therefore, it is unclear whether CUL4B affects the CSC state through participating in the EMT process. Indeed, we showed that increased CUL4B expression resulted in the induction of a stem‐like state, including upregulated expression of the stem cell markers *NANOG*, *SOX2*, *OCT4*, and *ID1*, as well as an increased CD44^high^/CD24^low^ population and mammosphere‐forming ability. Furthermore, consistent with our results in vitro, CUL4B significantly enhanced the tumor burden of NOD/SCID mice, possibly by stimulating an increase in the CSC population. We also found that high CUL4B expression was strongly correlated with poor prognosis in breast cancer patients. Therefore, CUL4B potentially promotes breast tumorigenesis by inducing a stem‐like state and enhancing their self‐renewal and tumor‐initiating capacities.

Although the molecular mechanism and functional network of the interaction between CRL4B and the four corepressor complexes remain to be further elucidated, our research focused on the biological significance of the physical interaction between CUL4B and the NuRD complex, and provided significant mechanistic insights into the CUL4B in breast cancer progression. These findings may shed light on how transcription factors enable the initial steps of metastasis, stimulation of EMT, physical dissemination, and finally development of a CSC phenotype.^[^ ^83^ ^]^ Notably, CRL4B was found positioned as a major transcriptional hub for regulating breast cancer progression, supporting the pursuit of CUL4B as a potential therapeutic target of breast cancer. Uncovering these patterns of epigenetic regulation by CRL4B can help us gain a deeper understanding of human cancers.

## 4 Experimental Section

##### Antibodies and Reagents

Antibodies used: *α*CUL4B, *α*HDAC1, *α*HDAC2, *α*RbAp46/48, and *α*‐fibronectin from Sigma‐Aldrich (St. Louis, MO); *α*DDB1, *α*MTA2, *α*MBD3, *α*SIN3A, *α*CtBP2, and *α*ER*α* from Santa Cruz Biotechnology (Dallas, TX); *α*SAP180, *α*SAP130, and *α*SAP30 from Bethyl (Montgomery, TX); *α*MTA3, *α*CtBP1, *α*CoREST, and *α*REST from Millipore (Billerica, MA); *α*MTA1 from Cell Signaling Technology (Danvers, MA); *α*ROC1, *α*NCoR, *α*HDAC3, *α*BHC80, *α*TBL1, *α*TBLR1 and *α*HIF1*α* from Abcam (Cambridge, UK); and *α*E‐cadherin, *αγ*‐catenin, and *α*N‐cadherin from BD Bioscience (San Jose, CA, USA). Control siRNA and siRNA for CUL4B were synthesized by Sigma‐Aldrich. shRNAs were obtained from Shanghai GenePharma (Shanghai, China). Protein A/G Sepharose CL‐4B beads were purchased from Amersham Biosciences (Little Chalfont, UK) and the protease inhibitor cocktail was obtained from Roche Applied Science (Penzberg, Germany).

##### Cell Culture and Transfection

The cell lines used were obtained from the American Type Culture Collection (ATCC, Manassas, VA). HEK293T cells, MCF‐7 cells, T‐47D cells, and Hs 578T cells were maintained in Dulbecco's modified Eagle's medium (DMEM) supplemented with 10% fetal bovine serum (FBS) and incubated in a humidified incubator with 5% CO_2_ at 37 °C. MDA‐MB‐231 and MDA‐MB‐453 cells were cultured in L‐15 medium supplemented with 10% FBS without CO_2_. Transfections were carried out using TurboFect Transfection Reagent (Thermo Fisher Scientific, Waltham, MA) and Lipofectamine RNAiMAX Reagent (Invitrogen, Carlsbad, CA) according to the manufacturer's instructions. Each experiment was performed in triplicate. For RNAi experiments, at least three independent siRNA/shRNA sequences were tested for each gene and the one with the highest efficiency was used. The shRNA sequences used are listed in Table S6 (Supporting Information 1).

##### Reporter Assay

MCF‐7 cells were transfected with different concentrations of the GAL4‐CUL4B expression plasmid together with the indicated GAL4‐luciferase reporter. Cells were treated with or without TSA. Luciferase activity was measured using a dual‐luciferase kit (Promega, Madison, WI) according to the manufacturer's instructions. Each experiment was performed in triplicate.

##### Immunoprecipitation and Western Blotting

Whole‐cell lysates were prepared by washing with cold PBS and incubating the cells in lysis buffer (50 × 10^‐3^ m Tris‐HCl, pH 8.0, 150 × 10^‐3^ m NaCl, and 0.5% NP40) for 30 min at 4 °C. This was followed by centrifugation at 12 000 × *g* for 15 min at 4 °C. For immunoprecipitation, 500 µg protein was incubated with specific antibodies or normal rabbit/mouse immunoglobin G (IgG; 2–3 µg) for 12 h at 4 °C with constant rotation. Next, 50 µL dynabeads protein G (Life Technologies, Carlsbad, CA) was added and the samples were further incubated for an additional 2 h at 4 °C. The beads were then washed five times in lysis buffer and the precipitated proteins were eluted from the beads by resuspending in 2× SDS‐PAGE loading buffer and boiling for 10 min. The resultant materials from immunoprecipitation, or cell lysates, were subjected to 10% SDS‐PAGE and transferred onto polyvinylidene fluoride membranes. For western blotting, membranes were incubated with the appropriate antibodies overnight at 4 °C, followed by incubation with secondary antibodies. Immunodetection was visualized using the enhanced chemiluminescence system (ECL; Thermo Fisher Scientific) according to the manufacturer's instructions.

##### Immunopurification and Mass Spectrometry

A stable MCF‐7 cell line expressing FLAG‐CUL4B was generated by transfecting cells with the corresponding FLAG‐tagged constructs for 48 h. Anti‐FLAG immunoaffinity columns were prepared using an anti‐FLAG M2 affinity gel (Sigma‐Aldrich) according to the manufacturer's instructions. FLAG peptide (0.2 mg mL^‐1^; Sigma‐Aldrich) was applied to the column to elute the FLAG protein complex. Fractions of the bed volume were collected, resolved using SDS‐PAGE, and silver stained. Proteins were then excised from the gel and subjected to liquid chromatography tandem mass spectrometry (LC‐MS/MS) sequencing and data analysis.

##### Fast Protein Liquid Chromatography

MDA‐MB‐231 cells were washed twice with cold PBS, scraped, and collected by centrifugation at 1500 × *g* for 5 min. Nuclear and cytoplasmic proteins were extracted using the Nuclear‐Cytosol Extraction Kit (Applygen Technologies, Beijing, China). The supernatant (nuclear fraction) was collected by centrifugation at 13 000 × *g* for 30 min at 4 °C. The protein concentration was determined using the BCA Pierce protein assay kit (Thermo Fisher Scientific) and MDA‐MB‐231 nuclear proteins (6 mg) were used for the FPLC assay. The proteins were concentrated to 1 mL using a Millipore Ultrafree centrifugal filter apparatus (10 kDa nominal molecular mass limit) and then applied to an 850 × 20 mm Superose 6 size exclusion column (Amersham Biosciences) that was equilibrated with buffer D (20 × 10^‐3^ m HEPES, pH 8.0, 10% glycerol, 0.1 × 10^‐3^ m EDTA, and 300 × 10^‐3^ m NaCl) containing 1 × 10^‐3^ m dithiothreitol and calibrated with protein standards (blue dextran, 2000 kDa; thyroglobulin, 669 kDa; ferritin, 440 kDa; catalase, 232 kDa; bovine serum albumin, 67 kDa; and RNase A, 13.7 kDa; Amersham Biosciences). The column was eluted at a flow rate of 0.5 mL min^‐1^ and fractions were collected.

##### GST Pull‐Down Assays

GST‐fused constructs were expressed in BL21 *Escherichia coli* and purified with 30 µL Glutathione Sepharose 4B beads. In vitro transcription and translation experiments were performed with rabbit reticulocyte lysate (TNT systems; Promega) according to the manufacturer's instructions. Then, GST fusion proteins were incubated with 5–8 µL in vitro transcribed/translated products in binding buffer (75 × 10^‐3^ m NaCl and 50 × 10^‐3^ m HEPES, pH 7.9) at 4 °C for 2 h in the presence of the protease inhibitor mixture. Finally, the beads were washed five times with binding buffer, resuspended in 30 µL 2× SDS‐PAGE loading buffer, and the proteins were detected by western blotting.

##### Real‐Time Quantitative RT‐PCR

Total cellular RNA was extracted with TRIzol reagent (Invitrogen) and used for first‐strand cDNA synthesis using the reverse transcription system.^[^ ^63^ ^]^ Relative quantitation of all transcripts was detected by real‐time RT‐PCR was performed using the Power SYBR Green PCR Master Mix on an ABI PRISM 7500 fast sequence detection system (Applied Biosystems, Foster City, CA). Relative quantitation of all transcripts was calculated using the comparative Ct method with *GAPDH* as an internal control. This assay was performed in triplicate. The primers used are listed in Table S4 (Supporting Information 1).

##### Chromatin Immunoprecipitation (ChIP), qChIP, and Re‐ChIP

ChIP experiments were performed with MDA‐MB‐231 cells and MCF‐7 cells. A total of 1 × 10^7^ cells were cross‐linked with 1% formaldehyde, sonicated, precleared, and incubated with 2–3 µg antibody for each reaction. Complexes were washed five times with low‐ and high‐salt buffers, and DNA was purified with the QIAquick PCR Purification Kit. qChIPs were performed using the TransStart Top Green qPCR supermix (TransGen Biotech, Shanghai, China). For Re‐ChIP assays, bead eluates from the first immunoprecipitation were incubated with 20 × 10^‐3^ m dithiothreitol (DTT) at 37 °C for 30 min and diluted at a ratio of 1:50 in ChIP dilution buffer (1% Triton X‐100, 2 × 10^‐3^ m EDTA, 150 × 10^‐3^ m NaCl, and 20 × 10^‐3^ m Tris‐HCl, pH 8.1) followed by reimmunoprecipitation with secondary antibodies. The final elution step was performed using 1% SDS in Tris–EDTA buffer (pH 8.0). The primers used are listed in Table S5 (Supporting Information 1).

##### ChIP Sequencing

MDA‐MB‐231 cells were maintained in L‐15 medium supplemented with 10% FBS. Approximately 5 × 10^7^ cells were used for each ChIP‐seq assay. Chromatin DNA was precipitated using either normal goat IgG (control) or polyclonal antibodies against CUL4B. DNA was purified with the Qiagen PCR purification kit (Qiagen, Hilden Germany) and in‐depth whole‐genome DNA sequencing was performed by the CapitalBio Corporation (Beijing, China). Raw sequencing image data were examined using the Illumina analysis pipeline, aligned to the unmasked human reference genome (UCSC GRCh37, hg19) using Bowtie2, and further analyzed by MACS. Enriched binding peaks were generated after filtering through the control IgG. The genomic distribution of CUL4B, MBD3, SIN3A, REST, and TBL1 binding sites was analyzed by ChIPseeker, an R package for ChIP peak annotation, comparison, and visualization. Pathway analysis was conducted based on the DAVID bioinformatics resource (https://david.ncifcrf.gov/).

##### Cell Invasion Assay

Transwell chamber filters were coated with Matrigel (BD Biosciences, Franklin Lakes, NJ). After infection with lentiviruses, 2.5 × 10^5^ MDA‐MB‐231 cells were suspended in 500 mL serum‐free L‐15 media, after which suspensions were placed in the upper chambers of the transwell. The chambers were then transferred to wells containing 500 µL media containing 10% FBS. After incubation for 18 h, cells in the top wells were removed by wiping the top of the membrane with cotton swabs. The membranes were then stained and the remaining cells counted. Five high‐powered fields were counted for each membrane.

##### In Vivo Metastasis in NOD/SCID Mice

MDA‐MB‐231 cells transfected to stably express firefly luciferase (Xenogen Corporation) were infected with lentiviruses carrying control shRNA, shCUL4B, empty vector, or the CUL4B expression construct. The cells were inoculated into the lateral tail veins (2 × 10^6^ cells) of 6 week old female SCID mice. Six animals per group were used for each experiment. For the orthotopic mouse of spontaneous breast cancer metastasis model, the mentioned indicated‐cells were suspended in a 1:1 mixture of PBS and Matrigel (BD Biosciences), then injected into the #4 mammary fat pads of 5 week old female NOD/SCID mice. Tumors were monitored weekly of the tumor length (*L*) and width (*W*), then removed after reaching a volume of 300 mm^3^. Tumor volume was calculated as *πLW* ^2^/6. Five animals per group were used for each experiment. For bioluminescence imaging, mice were abdominally injected with 200 mg g^‐1^ D‐luciferin in PBS. Fifteen minutes after injection, mice were anesthetized to image bioluminescence with a charge‐coupled device camera (IVIS; Xenogen). Bioluminescence images were obtained in a 15 cm field‐of‐view, binning (resolution) factor of 8, 1/*f* stop, open filter, and an imaging time of 30 s to 2 min. Bioluminescence from relative optical intensity was defined manually. Photon flux was normalized to background, which was defined based on the relative optical intensity drawn over a mouse not injected with luciferin. All animal handling and procedures were approved by the Institutional Animal Care and Use Committee of Cancer Hospital Chinese Academy of Medical Sciences.

##### In Vivo Tumorigenicity

For the tumor initiation study, MCF‐7 cells transfected to stably express firefly luciferase (Xenogen Corporation) were infected with lentiviruses carrying either an empty vector or the CUL4B expression construct. These cells were injected with Matrigel (BD Biosciences) into the #4 mammary fat pads of 6 week old female NOD/SCID mice at limiting dilutions of 2 × 10^6^, 1 × 10^6^, 1 × 10^5^, 1 × 10^4^, or 1 × 10^3^ cells. Eight mice were assayed per group. Tumors were visualized by bioluminescence imaging with the IVIS imaging system (Xenogen) at 4 weeks after injection. MDA‐MB‐231 cells stably transfected with control shRNA, shCUL4B were injected with Matrigel (BD Biosciences) into the #4 mammary fat pads of 6 week old female NOD/SCID mice at limiting dilutions of 5000, 1000, 500, 100 cells. Tumor growth was monitored for 2 months. Five mice were assayed per group.

##### Mammosphere Culture

A total of 5000 cells were plated in six‐well ultralow attachment plates in serum‐free DMEM‐F12 supplemented with 0.4% BSA, 20 ng mL^‐1^ bFGF, 10 ng mL^‐1^ EGF, and 5 µg mL^‐1^ insulin. Mammospheres were observed on day 5, which then increased in size and cell number until day 15.

##### Flow Cytometry

Indicated cells were digested, counted, and stained with the anti‐CD44‐FITC (BD Bioscience, New Jersey, USA) or anti‐CD133‐PE (Miltenyi Biotec, Bergisch Gladbach, Germany). To detect the expression levels of the enzyme aldehyde dehydrogenase (ALDH), experiments were performed using an ALDEFLUOR kit (StemCell, Cambridge, USA) according to the provided manual, single cells were suspended in assay buffer contain activated ALDEFLUOR reagent and incubated with or without diethylaminobenzaldehyde (DEAB). Samples were analyzed using FACSVerse (BD) flow cytometer. Data acquisition and analysis were performed using Flowjo v10.4 software.

##### Tissue Specimens and Immunohistochemistry

Samples were frozen in liquid nitrogen immediately after surgical removal and maintained at ‐80 °C until further analysis. Samples were fixed in 4% paraformaldehyde (Sigma‐Aldrich) at 4 °C overnight, embedded in paraffin, sectioned into 8 µm slices onto Superfrost‐Plus Slides, processed per standard protocols using 3,3′‐diaminobenzidine (DAB) staining, and monitored microscopically. All human tissues were collected using protocols approved by the Ethics Committee of Cancer Hospital Chinese Academy of Medical Sciences, and informed consent was obtained from all patients.

##### Statistical Analysis

Statistical analysis was performed using SPSS 19.0 software. Results are presented as the mean ± SD from at least three independent experiments unless otherwise noted. Comparisons were performed using a two‐tailed unpaired Student's *t*‐test for statistical analysis. *p* values < 0.05 were considered statistically significant, with *p < 0.05, **p < 0.01, ***p < 0.001, respectively. Correlation analysis was performed using the Pearson correlation test. Breast tumor datasets were downloaded from http://www.ncbi.nlm.nih.gov/geo and the GSE numbers are shown in the relevant figures. Data for Kaplan‐Meier survival analysis were obtained from http://kmplot.com/analysis, log‐rank tests were used for the statistical analysis.

## Conflict of Interest

The authors declare no conflict of interest.

## Author Contributions

W.H. and Y.W. conceived this project, wrote the manuscript, and secured funding. W.H., J.Z., M.H., T.Y., X.Y., P.W., S.L., Y.C. performed the experiments. W.H., J.Z., J.G., D.F., Y.Y. analyzed the data.

## Supporting information

Supporting Information

Supplementary Info

## Data Availability

We are willing to share these data: 1. The research data described in this manuscript. 2. The CUL4B ChIP‐Sequencing results: GSE124611 at http://www.ncbi.nlm.nih.gov/geo. 3. The data that supports the findings of this study in the Supporting Information of this article.
