# Supplementary material for: CUL4B Promotes Breast Carcinogenesis by Coordinating with Transcriptional Repressor Complexes in Response to Hypoxia Signaling Pathway
Source: Adv Sci (Weinh). 2021 Mar 16;8(10):2001515. doi: 10.1002/advs.202001515 (PMC8132058; doi:10.1002/advs.202001515)
Supplement: Supplementary file 1 — Supporting Information [file ADVS-8-2001515-s002.pdf]

© 2021 Wiley-VCH GmbH

ADVANCED SCIENCE
Open Access

Supporting Information

for *Adv. Sci.,* DOI: 10.1002/advs.202001515

CUL4B Promotes Breast Carcinogenesis by Coordinating with Transcriptional Repressor Complexes in Response to Hypoxia Signaling Pathway

*Wei Huang, Jingyao Zhang, Miaomiao Huo, Jie Gao, Tianshu Yang, Xin Yin, Pei Wang, Shuai Leng, Dandan Feng, Yang Chen, Yang Yang, and Yan Wang**

Copyright WILEY-VCH Verlag GmbH & Co. KGaA, 69469 Weinheim, Germany, 2018.

Supporting Information 1

CUL4B Promotes Breast Carcinogenesis by Coordinating with Transcriptional Repressor Complexes in Response to Hypoxia Signaling Pathway

*Wei Huang, Jingyao Zhang, Miaomiao Huo, Jie Gao, Tianshu Yang, Xin Yin, Pei Wang, Shuai Leng, Dandan Feng, Yang Chen, Yang Yang, and Yan Wang**

Dr. W. Huang, T. Yang, X. Yin, P. Wang, Prof. Y. Wang
Beijing Key Laboratory of Cancer Invasion and Metastasis Research
Advanced Innovation Center for Human Brain Protection
Department of Biochemistry and Molecular Biology
School of Basic Medical Sciences
Capital Medical University
Beijing 100069, China
E-mail: yanwang@cicams.ac.cn, yanwang@tmu.edu.cn

Dr. W. Huang, J. Zhang, M. Huo, Dr. J. Gao, Prof. Y. Wang
State Key Laboratory of Molecular Oncology
National Cancer Center/National Clinical Research Center for Cancer/Cancer Hospital
Chinese Academy of Medical Sciences and Peking Union Medical College
Beijing 100021, China

S. Leng, D. Feng, Y. Chen, Dr. Y. Yang, Prof. Y. Wang
Collaborative Innovation Center of Tianjin for Medical Epigenetics
Tianjin Key Laboratory of Medical Epigenetics
Key Laboratory of Immune Microenvironment and Disease (Ministry of Education)
Department of Biochemistry and Molecular Biology
School of Basic Medical Sciences
Tianjin Medical University
Tianjin 300070, China

The following files are included:

Supplementary Figures and Figure Legends (Figure S1-S8)

Supplementary Tables (Table S1-S6)

Supplementary Figures and Figure Legends:

Figure S1

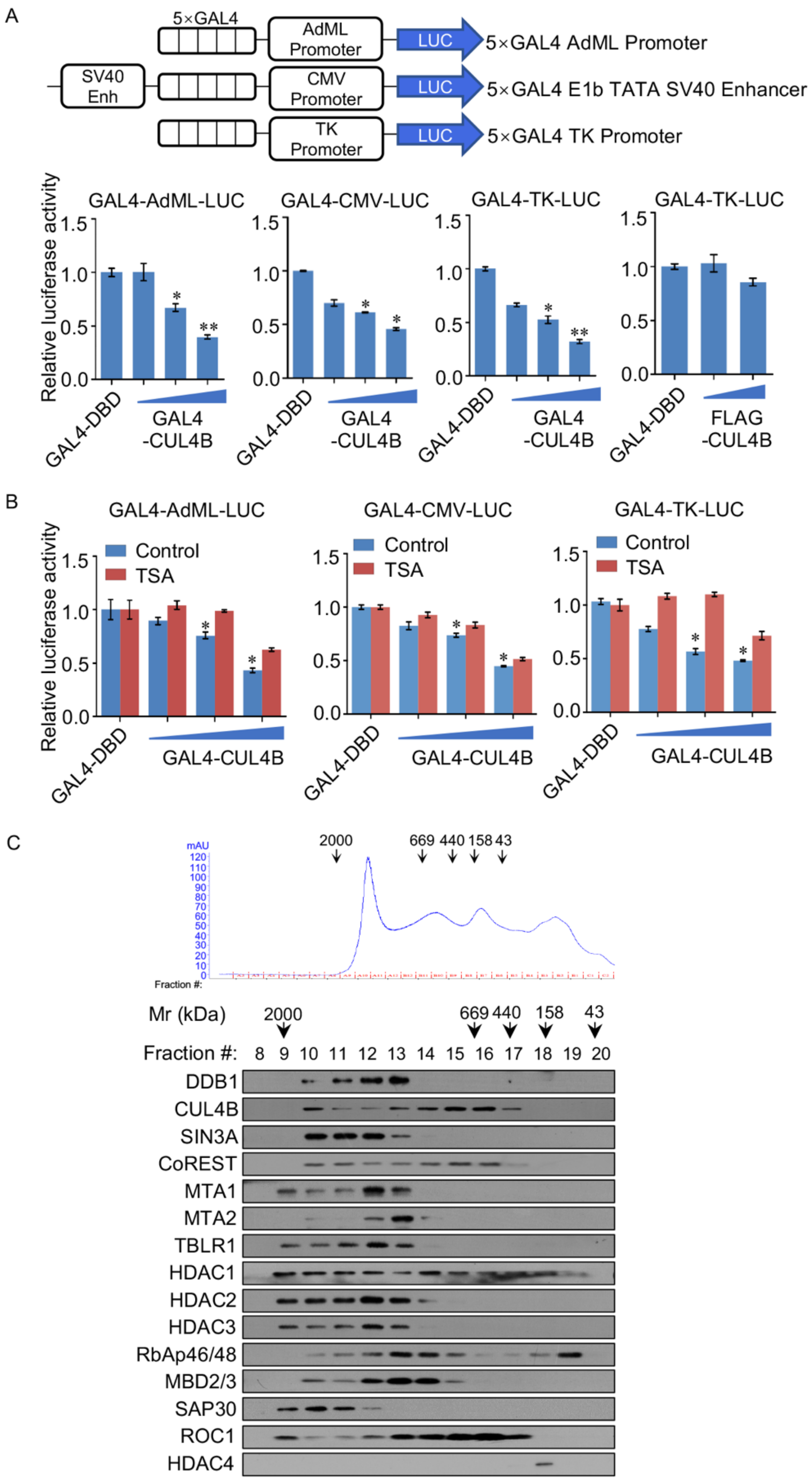

Figure S1. Cullin 4B-RING E3 Ligase is Physically Associated with HDAC-Containing Complexes

(A–B) Transcriptional repression by CUL4B. The schematic diagram shows the GAL4-luciferase reporters. For reporter assays, MCF-7 cells were transfected with the indicated GAL4-luciferase reporter, together with different doses of GAL4-CUL4B, FLAG-CUL4B expression construct, or with trichostatin A (TSA). Each bar represents the mean ± SD of three independent experiments. *$p < 0.05$, **$p < 0.01$. Student's *t*-test.

(C) Fast protein liquid chromatography assays in MDA-MB-231 cells. Nuclear extracts from MDA-MB-231 cells were concentrated and fractionated on Superose 6 size exclusion columns. The fractions were then analyzed by western blotting. The chromatographic elution profiles and elution positions of calibration proteins with known molecular masses (kDa) are shown.

Figure S2

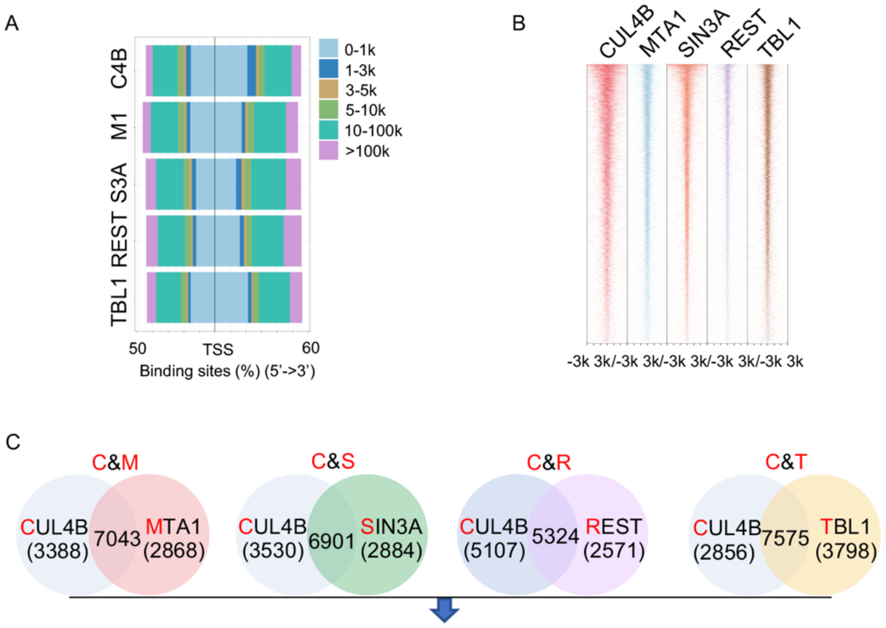

	C&M	C&S	C&R	C&T
Cell cycle	76	85	62	88
MAPK signaling pathway	107	106	76	124
Focal adhesion	77	81	62	98
p53 signaling pathway	39	45	34	46
Hypoxia signaling pathway	48	47	30	53
TGF-beta signaling pathway	41	42	26	53
Wnt signaling pathway	57	61	35	65
Signaling pathways regulating pluripotency of stem cells	56	56	47	70
Estrogen signaling pathway	38	38	36	46
Notch signaling pathway	21	25	16	21

Figure S2. Identification of Genome-wide Transcription Targets of the CRL4B Complex

(A) The distribution of binding loci relative to the transcriptional start sites (TSSs) of CUL4B, MTA1, SIN3A, REST, and TBL1. C4B, CUL4B; M1, MTA1; S3A, SIN3A.

(B) Heatmap of the promoter peaks of CUL4B, MTA1, SIN3A, REST, and TBL1.

(C) Venn diagrams of overlapped promoters bound by CUL4B/MTA1, CUL4B/MTA1, CUL4B/SIN3A, CUL4B/REST, and CUL4B/TBL1; overlapping signaling pathways were functionally clustered using DAVID.

Figure S3

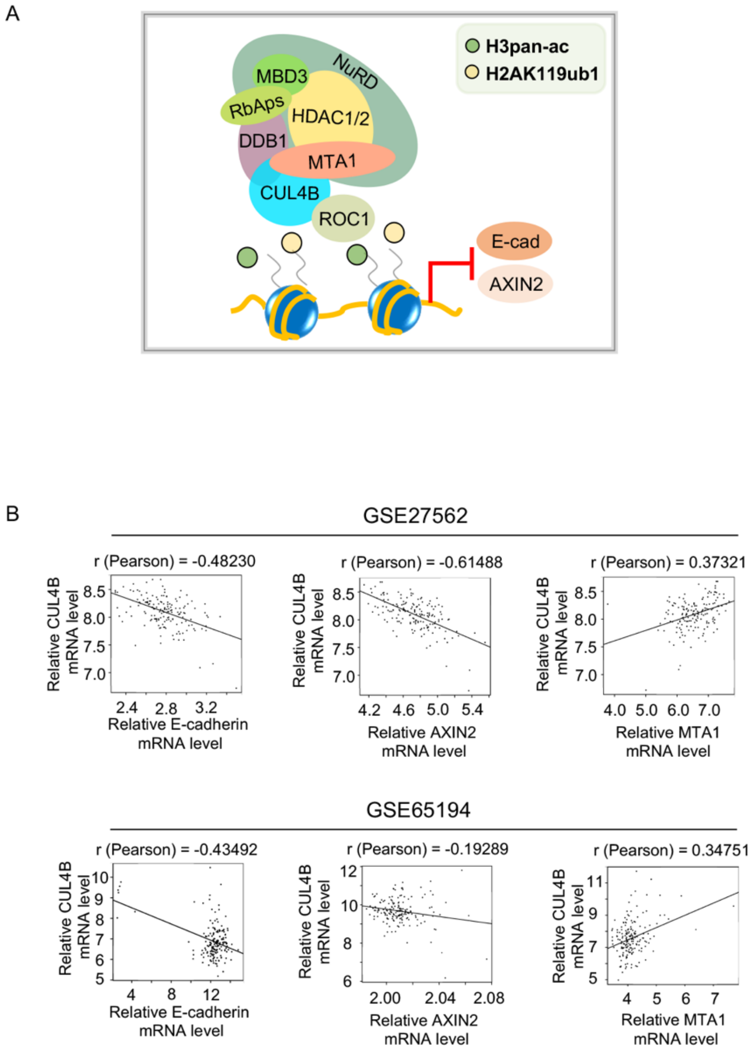

Figure S3. CRL4B is Physically Associated with the NuRD(MTA1) Complex

(A) Graphic model of the interaction between the CRL4B complex and the NuRD complex. E-cad, E-cadherin.

(B) Analysis of public datasets for the expression of E-cadherin, AXIN2, MTA1, and CUL4B in breast carcinomas. The relative levels of E-cadherin, AXIN2, and MTA1 were plotted against that of CUL4B.

Figure S4

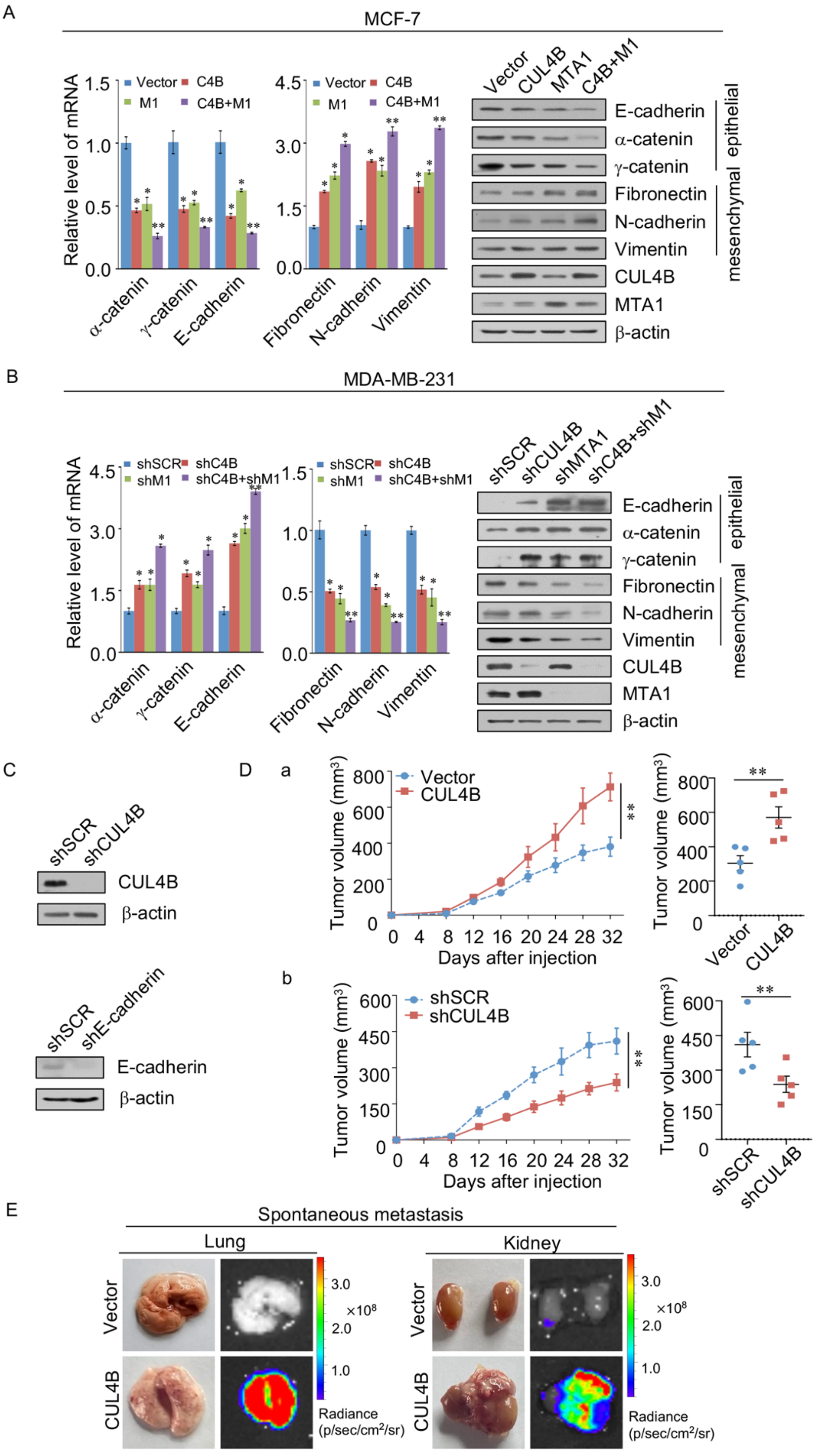
A
MCF-7
Relative level of mRNA
Vector
C4B
M1
C4B+M1
α-catenin
γ-catenin
E-cadherin
Fibronectin
N-cadherin
Vimentin
CUL4B
MTA1
β-actin
epithelial
mesenchymal
B
MDA-MB-231
shSCR
shC4B
shM1
shC4B+shM1
shCUL4B
shMTA1
C
shE-cadherin
D
a
Tumor volume (mm3)
Days after injection
b
E
Spontaneous metastasis
Lung
Kidney
Radiance
(p/sec/cm2/sr)
×10^8

Figure S4. The CRL4B/NuRD (MTA1) Complex Promotes the Invasion of Breast Cancer Cells

(A) RT-PCR analysis of the mRNA expression patterns of epithelial and mesenchymal markers (left) and immunoblotting to assess the indicated protein levels (right) in MCF-7 cells stably overexpressing CUL4B and/or MTA1. C4B, CUL4B; M1, MTA1. Error bars represent mean ± SD of three independent experiments. *$p < 0.05$, **$p < 0.01$. Student's *t*-test.

(B) MDA-MB-231 cells were transfected with shRNA against CUL4B or/and MTA1. The expression of epithelial and mesenchymal markers was assessed by RT-PCR (left) and western blotting (right) in these cells. Error bars represent mean ± SD of three independent experiments. *$p < 0.05$, **$p < 0.01$. Student's *t*-test.

(C) MDA-MB-231 cells infected with lentiviruses carrying shCUL4B or in combination with lentiviruses carrying shE-cadherin. The efficiency of protein knockdown was verified by western blotting.

(D) Tumor growth curve following CUL4B overexpression (a) or knockdown (b) in MDA-MB-231 cells were monitored over the indicated time period (left panels). Tumor volumes at the endpoint were shown in the right panels. Data are presented as mean ± SEM. *$p < 0.05$, **$p < 0.01$. Student's *t*-test.

(E) Spontaneous metastases on multiple organs such as lungs, kidneys were verified by quantitative bioluminescence imaging 3 weeks after removal of the primary tumors.

Figure S5

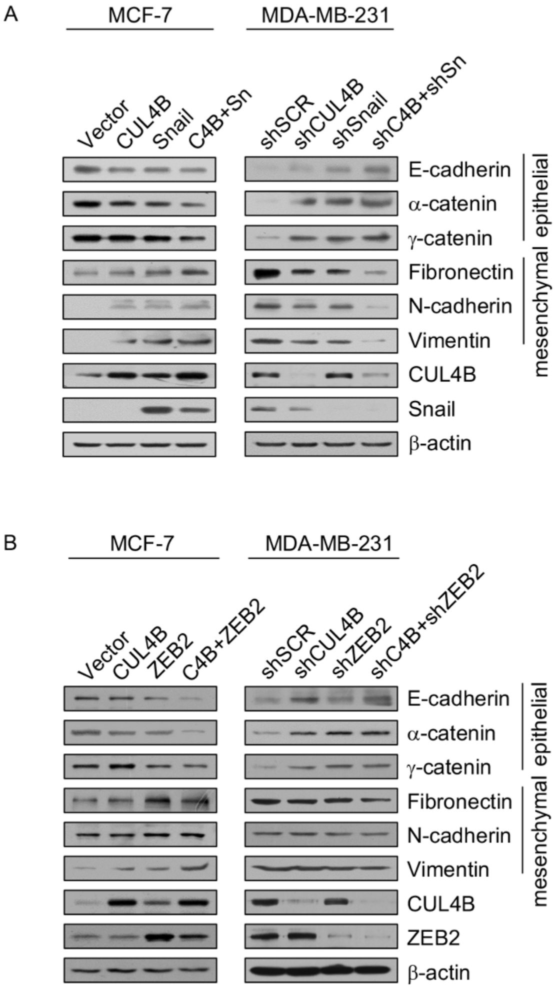

Figure S5. The CRL4B/NuRD(MTA1) Complex was Recruited by the Transcription Factors Snail/ZEB2 to Promote Breast Cancer Metastasis

(A) Epithelial and mesenchymal marker changes were assessed by western blotting in CUL4B- and/or Snail-expressing MCF-7 cells, or in CUL4B- and/or Snail-knockdown MDA-MB-231 cells. C4B, CUL4B; Sn, Snail.

(B) Epithelial and mesenchymal marker changes were assessed by western blotting in CUL4B- and/or ZEB2-expressing MCF-7 cells, or in CUL4B- and/or ZEB2-knockdown MDA-MB-231 cells. C4B, CUL4B.

Figure S6

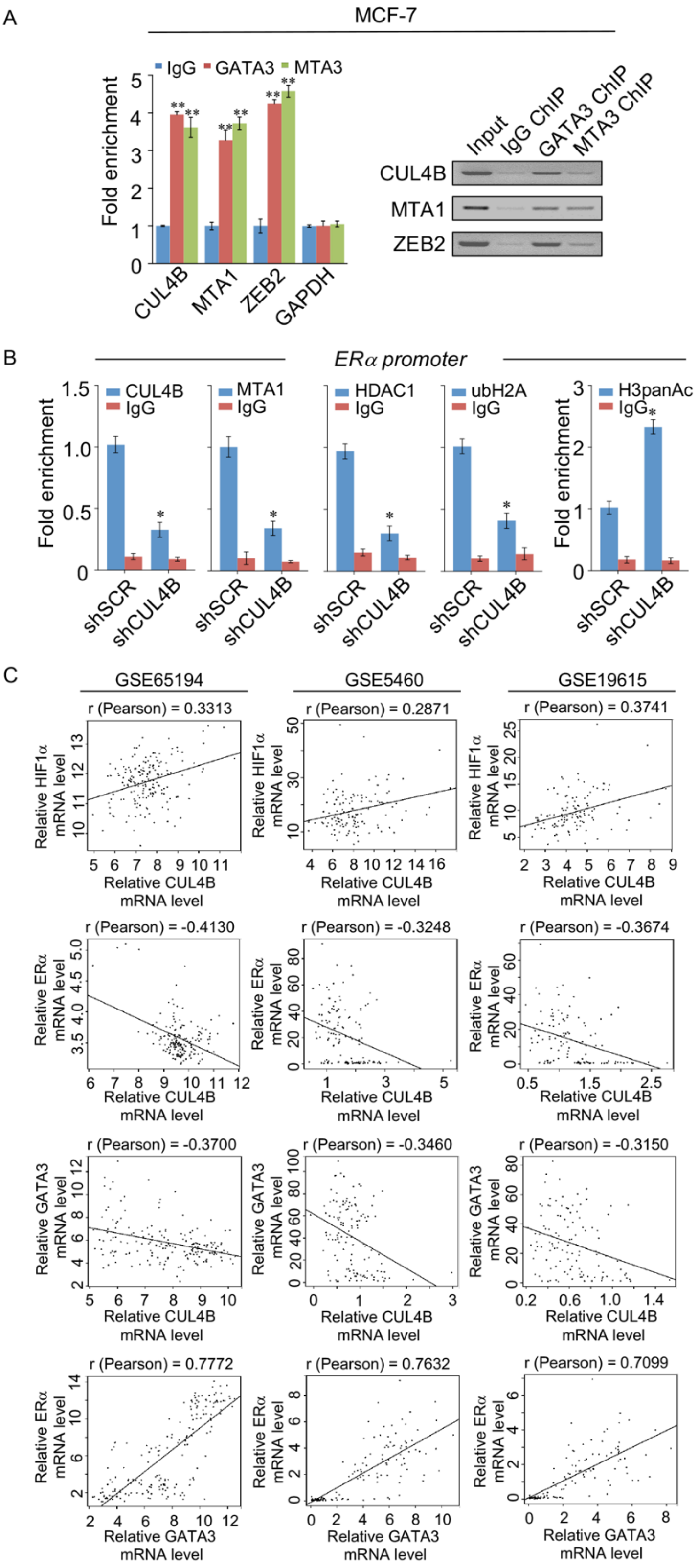
A
MCF-7
IgG GATA3 MTA3
Fold enrichment
CUL4B
MTA1
ZEB2
GAPDH
Input
IgG ChIP
GATA3 ChIP
MTA3 ChIP
B
ERα promoter
CUL4B
MTA1
HDAC1
ubH2A
H3panAc
IgG
shSCR
shCUL4B
C
GSE65194
GSE5460
GSE19615
r (Pearson) = 0.3313
r (Pearson) = 0.2871
r (Pearson) = 0.3741
r (Pearson) = -0.4130
r (Pearson) = -0.3248
r (Pearson) = -0.3674
r (Pearson) = -0.3700
r (Pearson) = -0.3460
r (Pearson) = -0.3150
r (Pearson) = 0.7772
r (Pearson) = 0.7632
r (Pearson) = 0.7099
Relative HIF1α mRNA level
Relative ERα mRNA level
Relative GATA3 mRNA level
Relative CUL4B mRNA level
Relative GATA3 mRNA level

Figure S6. CUL4B Depletion Abolishes Hypoxia/Wnt/Notch-induced EMT and CUL4B is Negatively Regulated by the Estrogen-ERα-GATA3 Axis

(A) qChIP (upper panels) and ChIP assays (lower panels) in MCF-7 cells with antibodies against GATA3 or MTA3 at the *CUL4B*, *MTA1*, and *ZEB2* promoters. Error bars represent mean ± SD of three independent experiments. **p* < 0.05, ***p* < 0.01. Student's *t*-test.

(B) MDA-MB-231 cells were transfected with shSCR and shRNAs targeting CUL4B. qChIP analysis of the recruitment of the *ERα* promoter was performed using the indicated antibodies. Purified rabbit IgG was used as a negative control. ubH2A, H2AK119ub1. Error bars represent mean ± SD of three independent experiments. **p* < 0.05, ***p* < 0.01. Student's *t*-test.

(C) Analysis of public datasets (GEO: GSE65194, GSE5460, GSE19615) for the expression of CUL4B, HIF1α, ERα, and GATA3.

Figure S7

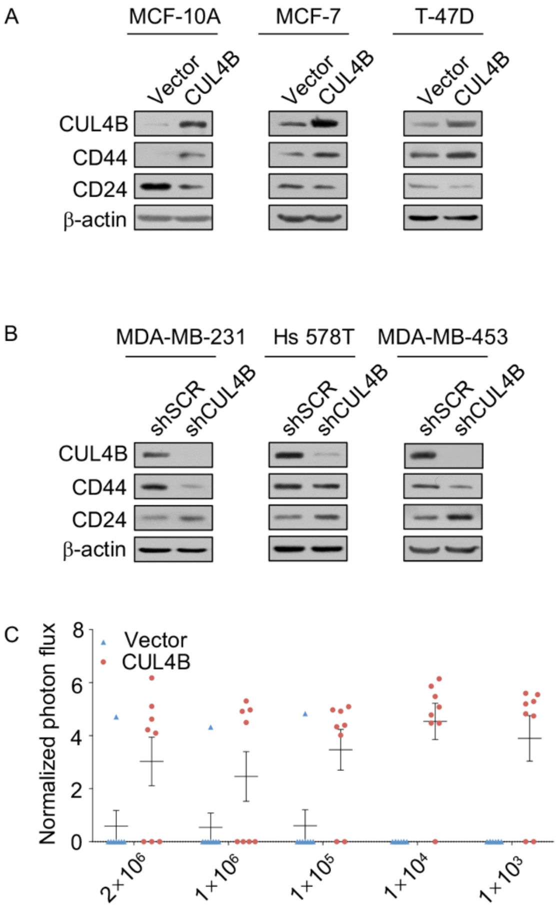

Figure S7. CUL4B Promotes the Growth of Breast Tumor Xenografts in NOD/SCID Mice by Upregulating the Breast Cancer Stem Cell Population

(A) Western blot analysis of CD44 and CD24 expression in CUL4B-overexpressing MCF-7, T-47D, or MCF-10A cells.

(B) Western blot analysis of CD44 and CD24 expression in CUL4B-knockdown MDA-MB-231, Hs 578T, or MDA-MB-453 cells.

(C) Tumorigenicity was determined by injecting Vector- or CUL4B-expressing MCF-7 cells at various dilutions. The endpoint photon flux of each group was statistically analyzed. (n = 8 in each group).

Figure S8

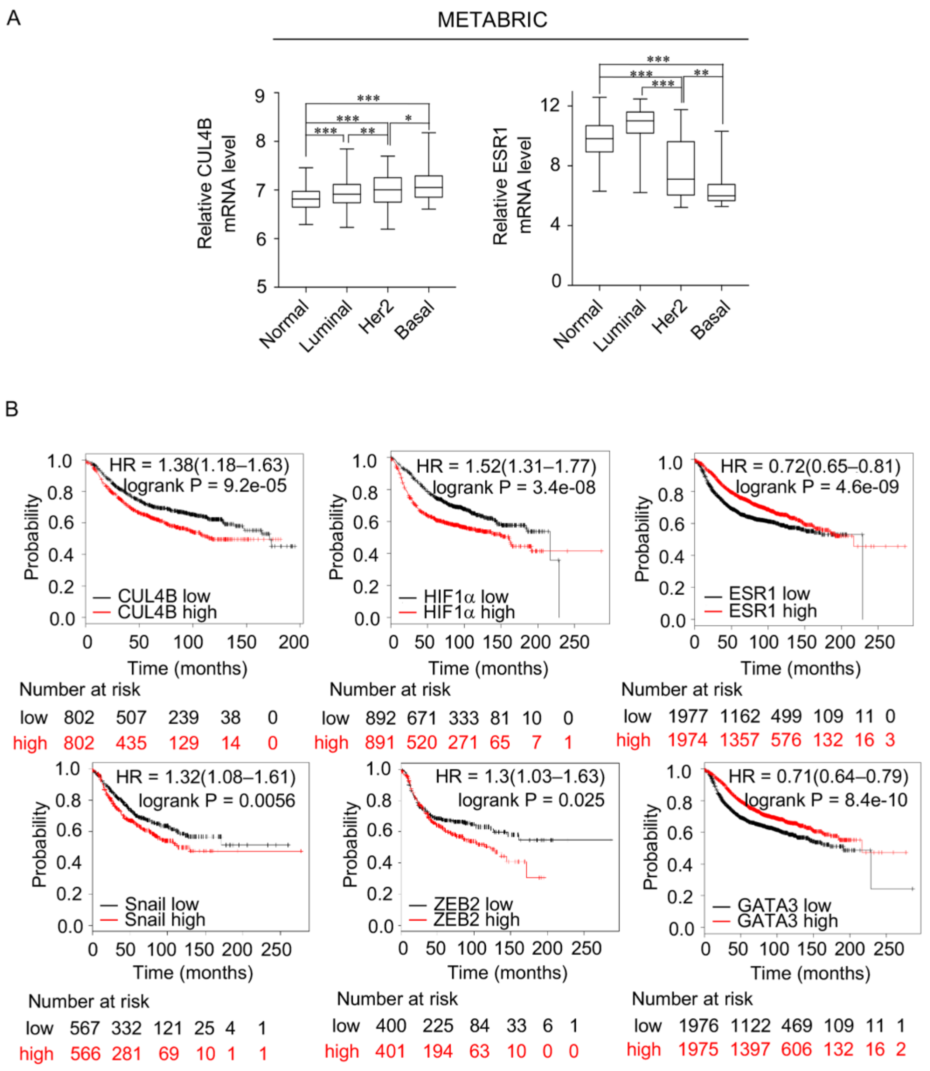

Figure S8. CUL4B is Upregulated in Breast Cancer and is a Potential Cancer Biomarker

(A) Bioinformatics analysis of METABRIC public datasets for the expression of CUL4B and ERα in several molecular subtypes of breast cancer. Data are presented as mean ± SEM. *$p < 0.05$, **$p < 0.01$, ***$p < 0.001$. Student's *t*-test.

(B) Kaplan-Meier survival analysis of the relationship between survival time and CUL4B/HIF1α/Snail/ZEB2/ERα/GATA3 expression signatures in breast cancer using the KM-plot online tool (http://kmplot.com/analysis/).

Table S1, related to Figure 1:

Mass Spectrometry Analysis of CUL4B-containing Protein Complex

Band	Identified proteins	Peptides
280kDa	FLNA	AFGPGLQGGSAGSPAR<br>AGQSAAGAAPGGGVDTR<br>ANLPQSFQVDTSK<br>DAGEGLLAVQITDPEGKPK<br>EATTEFSVDAR<br>EGPYSISVLYGDEEVPR<br>GKLDVQFSGLTK
220kDa	E1F4G1	ALPSEELNR<br>GPAGLGPR
140kDa	USP7	ITDFENR<br>LLEIVSYK<br>VLLDNVENK<br>FDDDVVSR
130KDa	CAND1	AADIDQEVK<br>ALTLIAGSPLK<br>HTVDDGLDIR<br>IGEYLEK<br>NGEVQNLAVK<br>TLEDPDLNVR<br>VYPSSLSK
130kDa	DDB1	LEIYVVTAEGLR<br>VIPLDRDNKELK<br>FLYGCQAPTICFVYQDPQGR<br>QSTIVCHNR<br>YLLGDMEGRLFMLLLEK<br>QGQGQLVTCSGAFK<br>NGIGIHEHASIDLPGIK<br>LPSFELLHK
120kDa	USP15	ADTIDTIEK<br>AEGGAADLDTQR<br>SDIATLLK<br>SYAELIK
120kDa	KIF11	AVDQHNAEAQDIFGK<br>SKVEETTEHLVTK<br>NILNKPEVNQK<br>GKNIQVVVR<br>ILQDSLGGR
110kDa	OGT	IIFSPVAPK<br>SDLGNLLK<br>TIIVTTR<br>EQGNIEEAVR
107kDa	GANAB	AFFAGSQR<br>IDELEPR<br>NLGLYVK<br>VTEGGEPYR

105kDa	CUL4B	AFGSTIVINPEK<br>AGNKEATDEELEK<br>DIMIQFK<br>DKENPNQYNYIA<br>DVFEAFYK<br>EAFETFINK<br>EAVEAIQNSTSIK<br>EDSLDSVLFLK<br>FPVKPADLK<br>GLNNLLDENR<br>LEGMFK<br>QATGIEDGELR<br>QYQIDAAIVR
103kDa	DCAF5	EDPTDTPATDSSR<br>SIVNQVR
100kDa	MCM3	APAGQLPR<br>DAQPSFSAEDIAK<br>ELISDNQYR<br>LIVNVNDLR<br>VQVVGTYR
100kDa	HSP90B1	DISTNYYASQK<br>EAESSPFVER<br>GLFDEYGSK<br>LGVIEDHSNR<br>LSLNIDPDAK<br>SGTSEFLNK
95kDa	EEF2	AGIIASAR<br>FSVSPVVR<br>GEGQLGPAER<br>GGGQIIPTAR<br>SDPVVSYR<br>VNFTVDQIR
80kDa	MTA1	RPYKPIALR (MTA1 Specific)<br>QIDQFLVVAR<br>KPLEAVLR (MTA1 Specific)<br>CSVTLLNETESLK<br>DITLFHAMDTLHK
76kDa	SFPQ	AELDDTPMR<br>FATHAAALSVR<br>FGQGGAGPVGGQGPR<br>ISDSEGFK
75kDa	DDX3X	VGSTSENITQK<br>DLMAcAQTGSGK<br>SPILVATAVAAR<br>QSSGASSSSFSSSR
73kDa	PRMT5	AAMAVGGAGGSR<br>EFIQEPAK<br>GPLVNASLR<br>SDLLLSGR<br>LYAVEK

67kDa	MTA3	RVGDYVYFENSSSNPYLIRR<br>KTLLADKGEIRV<br>RPFVAINYAAIR<br>QIDQFLVVAR<br>SLTSIIEYYYMWK
66kDa	KRT1	AEAESLYQSK<br>AQYEDIAQK<br>FLEQQNQVLQTK<br>IEISELNR<br>SLVNLGGSK
61kDa	DCAF11	GSFSLGEQSR<br>NSSSAGSGSGDPSEGLPR<br>YNPPVDATPDTR<br>YLISNSK
56kDa	GPS1	SPPREGSQGELTPANSQSR<br>MALSFVQR<br>AESTPEIAEQR
55kDa	HDAC1	KQQTDIAVNWAGGLHHAK<br>RMTHNLLLNYGLYRK<br>IKQRLFENLR
55kDa	HDAC2	RQQTDMAVNWAGGLHHAKK<br>FEFCQLSTGGSVAGAVK<br>YGEYFPGTGDLRDIGAGK
50kDa	HDAC3	TVAYFYDPDVGNFHYGAGHPMK<br>PYQASQHDMCR<br>VSPTNMQGFTK
48kDa	RbAp48	KHPSKPDPSGECNPDLR<br>KIEIEIKINHEGEVNRA<br>IGEEQSAEDAEDGPPELLFIHGGHTAK
47kDa	CTBP2	VQSVEQIR
42kDa	MEP50	DSVFLSCSEDNR<br>SDGALLLGASSLSGR<br>VWDLAQQVVLSSYR<br>FCKYEHDDIVSTVSVLSSGTQAVSGSK
37kDa	COPS5	LEQSEAQLGR<br>VAVVIDPTR
33kDa	MBD3	RYLGGSMDLSTFDFRT<br>KLSGLNAFDIAEELVKT<br>YDSSNQVK
30kDa	SAP30	AQLVEIVGcHFR<br>HLYICDYHK<br>DTLTYFIYSVK
23kDa	HP1	VVEKVLDR<br>SNFSNSADDIK<br>CPQIVIAFYEER

Table S2, related to Figure 5:

Mass Spectrometry Analysis of Snail-containing Protein Complex

Band	Identified proteins	Peptides
130kDa	DDB1	ALVSEWKEPQAK<br>YLLGDMEGR<br>EMLGGEIIPR<br>PTVIYSSNHK<br>TVPLYESPR
105kDa	CUL4B	RPSTPTINVLE<br>GLNNLLDENR<br>LITYLDQTTQK
80kDa	MTA1	CSVTLLNETESLK<br>YLETHPRPPKPDPVK<br>LPEASQSPLVLK<br>DEMEEWSASEANLFEEALEK
75kDa	MTA2	LNPADAPNPVVFVATK<br>RIEELNK<br>AVGTFAR<br>PEAQSLSPYTTSANR
55kDa	HDAC1	SIRPDNMSEYSK<br>IACEEEFSDSEEEGEGGRK<br>VMTVSFHK<br>ANAEEMTKYHSDDYIK<br>IRMTHNLLLNYGLYR<br>MTHNLLLNYGLYR
55kDa	HDAC2	LGCFNLTVK<br>ATAEEMTKYHSDEYIK<br>QQTDMAVNWAGGLHHAKK
50kDa	HDAC3	LALTHSLVLHYGLYK<br>VLYIDIDIHHGDGVQEAFYLTDR
48kDa	RbAp48	HPAKPDPSGECNPDLR<br>KGTGDSK
33kDa	MBD3	WECPALPQGWER<br>YLGGSMDLSTFDFR<br>LEEALMADMLAHVEELAR
30kDa	Snail	VAELTSLSDEDSGK<br>AFSRPWLLQGHVR<br>YCNKEYLSLGALK

Table S3, related to Figure 5:

Mass Spectrometry Analysis of ZEB2-containing Protein Complex

Band	Identified proteins	Peptides
137kDa	ZEB2	KSIIDYTLEK<br>KMIENHNISTPFSCQFCK<br>KPYQCDKCGKR<br>NANCTSDFEEYFAK
130kDa	DDB1	SAEDLNLLIAK<br>IEVQDTSGGTTALR<br>NISVASCNSSQVVVAVGR
105kDa	CUL4B	GLNNLLDENR<br>SNRQKILER<br>LQWQSTLGHCVLK<br>LYAAEGQK
80kDa	MTA1	QIDQFLVVAR<br>LCASCWTYWK<br>YLETHPRPPK<br>AISALVPQGGPVLCR<br>DFTDIQQDFLPWK
67kDa	MTA2	RPNLPLK<br>KAAKTPLKIHPLVR<br>QVYIPTYTK<br>AVGTFAR
55kDa	HDAC1	VEPFVCAQCR<br>TDFTPHWK<br>ALQQEQEIEQR<br>YGEYFPGTGDLRDIGAGK<br>LHISPSNMTNQNTNEYLEK
55kDa	HDAC2	TFNLPLLMLGGGGYTIR<br>SIRPDNMSEYSK<br>YYAVNFPMR
50kDa	HDAC3	YGNYFFPGTGDMYEVGAESGR<br>DGIDDQSYK<br>MLNHAPSVQIHDVPADLLTYDR
48kDa	RbAp48	KTPSSDVLVFDYTKH<br>KEAAFDDAVEER
33kDa	MBD3	TLLADQGEIR<br>SGLSAGHR<br>LSGLNAFDIAEELVK

Table S4:

The Primers used in quantitative real-time PCR (qPCR)

Gene	Strand	Sequence
E-cadherin	F	CATTTCTTGGTCTACGCCTG
E-cadherin	R	GAGAGGAGTTGGGAAATGTG
α-catennin	F	AGCTGAAAGTTGTGGAAGAT
α-catennin	R	CCAACATCTTTCAATTCCTGTTG
γ-catennin	F	GGACAAGAACCCAGACTACC
γ-catennin	R	GTGGCATCCATGTCATCTCC
Fibronectin	F	CCATCCATTGATTTAACCAACTT
Fibronectin	R	TACCAGGCAGGAGATTTGTTAA
N-cadherin	F	CACTGCTCAGGACCCAGAT
N-cadherin	R	TAAGCCGAGTGATGGTCC
Vimentin	F	ATTGAGATTGCCACCTACAG
Vimentin	R	ATCCAGATTAGTTTCCCTCAG
GAPDH	F	GTCAACGGATTTGGTCGTAT
GAPDH	R	GAACATGTAAACCATGTAGTTGA
OCT4	F	ATCACCCTGGGATATACACAG
OCT4	R	CTGCTTTGCATATCTCCTGA
SOX2	F	GCCTGGGCGCCGAGTGGA
SOX2	R	GGGCGAGCCGTTCATGTAGGTCTG
ID1	F	GAGGAATTACGTGCTCTGTG
ID1	R	GTCTCTGGTGACTAGTAGGTG
C-MYC	F	AAACTTGAACAGCTACGGAAC
C-MYC	R	ATTTGAGGCAGTTTACATTATGG
NANOG	F	TCTGGACACTGGCTGAATCCT
NANOG	R	CGCTGATTAGGCTCCAACCAT
AXIN2	F	AGGTTCTGGCTATGTCTTTG
AXIN2	R	AATTCCATCTACACTGCTGTC
ERα	F	GGGAAGTATGGCTATGGAATCTG
ERα	R	TGGCTGGACACATATAGTCGTT
ATM	F	TTGATCTTGTGCCTTGGCTAC
ATM	R	TATGGTGTACGTTCCCCATGT
p21	F	TGTCACTGTCTTGTACCCTT
p21	R	AGTGGTAGAAATCTGTCATGCT
p18	F	GAACAAGGGACCCTAAAGAA
p18	R	TCGGGATTTCCAAGTTTCAT
BDNF	F	CTACGAGACCAAGTGCAATCC
BDNF	R	AATCGCCAGCCAATTCTCTTT

LAMA5	F	CTCTGGCTGGATTATGTACT
LAMA5	R	GGGCTCCGTTGTTATAGAAG
ROBO3	F	ATATTACAACGAAGCGGGAAT
ROBO3	R	AGATCTCCTGAGGGATGTTG
AKT1	F	TCCTCCTCAAGAATGATGGCA
AKT1	R	GTGCGTTCGATGACAGTGGT
TRAF4	F	GACTATGCCAAGATCTACCC
TRAF4	R	AGGGAATGACATTGAAGCTG
TSC1	F	CAACAAGCAAATGTCGGGGAG
TSC1	R	CATAGGGCCACGGTCAGAA
CUL4B	F	GGCAACTGGAATAGAGGATG
CUL4B	R	TGTTCTTCAACCGTTTCTTTC
DDB1	F	ATCATCAAGCAAAGCACGATT
DDB1	R	ATCAAGGTATGTCAAGCACTC
MTA1	F	GGAAGACCACCGACAGATAC
MTA1	R	GGATTTGGCTTGTTATAGTTGGG
HDAC1	F	GAAATCTATCGCCCTCACAA
HDAC1	R	GTAGACAACTGACAGAACTCAA
GATA3	F	ATCTGTCTTGTCCCTATTCC
GATA3	R	ATCACTAACACAGAACACGA
MTA3	F	CATCACCTTGTTTCACGCTA
MTA3	R	CCATTCCTCCATTTCATCTCT

Table S5-1:

The Primers used in qChIP Assays

Gene	Strand	Sequence
AXIN2	F	AAAGTCCTCCAAGCCCAAAT
AXIN2	R	ATCGCAAGAACTGCAAGCAA
ERα	F	GAACCAATATCCCAACCCTAATACT
ERα	R	CAGCTAATGAATTCTGAACTGAAGG
E-cadherin	F	TTTGCTGACTCACTAACCCAT
E-cadherin	R	AGGCTGGTCTGGAACTCCT
GAPDH	F	AGCCACATCGCTCAGACACC
GAPDH	R	CCCATACGACTGCAAAGACCC
ATM	F	AGAACCTCCGAATGACGAAGA
ATM	R	GGCTATGCTGGACAGAAGGA
p21	F	AGGCTGTGGCTCTGATTGG
p21	R	GTCTCCTGTCTCCTACCATCC
p18	F	GAGAACTTCGGCAACCAAGAA
p18	R	AGGACTGGAAACTGCGAAATAG
BDNF	F	GATAAGATCAGTGAAAGGCAGAG
BDNF	R	CTGGGTGACCAGAGTGAAACT
LAMA5	F	CGGAGCCCAAGGGAAGTA
LAMA5	R	AGGACGCCTGGCAAACAG
ROBO3	F	CCAGGAAGGACGACTGCTAT
ROBO3	R	GGAACCAAGGATTCTAGGACAA
AKT1	F	CTGACGGACTTGTCTGAACCT
AKT1	R	CTGCCGGTCTGAATCTGGT
TRAF4	F	GAGCCGAGATCGCACCACT
TRAF4	R	GGAGATAGAGTCCCGGTAAGCA
TSC1	F	CGTCCAACCCACATCGTC
TSC1	R	GCTGTTTACCTCACAGTCCCT
CUL4B	F	AGCATTCTTCTTCCTTTGGGTAC
CUL4B	R	CTGGGTCACATCCTTACAGTTCA
MTA1	F	CACGCCATTCTTCTGCCTCAG
MTA1	R	TCGAGACCATCCTGGCTAACAC
ZEB2	F	GTGAGGTCTCCCCGAGGTGT
ZEB2	R	ATTAGGGTGTGTGCATGATGAAT

Table S5-2:

The Primers used in ChIP Assays

Gene	Strand	Sequence
AXIN2	F	CATATTAGGCAGGCTGGTCTC
AXIN2	R	CACAACCTTCTCTTCTCCGTTA
p21	F	TCCTCACATCCTCCTTCTTCAG
p21	R	TAGCCACCAGCCTCTTCTATG
BDNF	F	AGATTTAGTTGTTAGCGGAATAGAC
BDNF	R	ATTCAAAGGCAACCTACCTCC
TSC1	F	TAGGCTCTTGCTCCTTTGG
TSC1	R	GCGAGGCTGAGTATGATGG
E-cadherin	F	GAGGGTCACCGCGTCTATG
E-cadherin	R	ACTTCCGCAAGCTCACAGG
CUL4B-800	F	AGTTGGGTTTCTGTCACTTGC
CUL4B-800	R	GGTGGTGGGTGTATTGATGTT
CUL4B-HBS-1	F	AACAGGAGCCTAGACTTCGG
CUL4B-HBS-1	R	ATGGGACTTAAACAGATAAGCC
CUL4B-HBS-2	F	AGTAAAGAGTATCGAAAAGCCAA
CUL4B-HBS-2	R	CATTTCTTGGATTCTCAATGTGT
VEGF	F	TTTCCAAAGCCCATTCCC
VEGF	R	AGACGCTGCTCGCTCCAT
MTA1	F	TGCTGGGATTACAGGCGTGAG
MTA1	R	ACCAGTGAACAAGGTGGATTGC

Table S6:

shRNA sequences

shSCR	TTCTCCGAACGTGTCACGT
shCULB	GGATTCATTGGATAGCGTTCT
shMTA1	GGCTAACTTATTCCGAGAATG
shSIN3A	GGAAATTCCCTGAGTTGTTTA
shCoREST	ACAACCACCTAAGTGATAATA
shHDAC3	CCTGCATTACGGTCTCTATAA
shDDB1	TCCACTAGATCGCGATAATAA
shHDAC1	GCTGCTCAACTATGGTCTCTA
shZEB2	CCCGAAACGATACGAGATGAA
shSnail	GCAAATACTGCAACAAGGAAT
LV-16	TTCTCCGAACGTGTCACGT
shEcadherin	GCAGACCTTCCTCCCAATACA
shMTA3	GCTTTCTTCCTTCATACTACA
shGATA3	CATCCAGACCAGAAACCGAAA
